# Complex genetics cause and constrain fungal persistence in different parts of the mammalian body

**DOI:** 10.1093/genetics/iyac138

**Published:** 2022-09-14

**Authors:** Martin N Mullis, Caleb Ghione, Michael Lough-Stevens, Ilan Goldstein, Takeshi Matsui, Sasha F Levy, Matthew D Dean, Ian M Ehrenreich

**Affiliations:** Molecular and Computational Biology Section, Department of Biological Sciences, University of Southern California, Los Angeles, CA 90089, USA; Molecular and Computational Biology Section, Department of Biological Sciences, University of Southern California, Los Angeles, CA 90089, USA; Molecular and Computational Biology Section, Department of Biological Sciences, University of Southern California, Los Angeles, CA 90089, USA; Molecular and Computational Biology Section, Department of Biological Sciences, University of Southern California, Los Angeles, CA 90089, USA; Joint Initiative for Metrology in Biology, Stanford University, Stanford, CA 94305, USA; SLAC National Accelerator Laboratory, Menlo Park, CA 94025, USA; Department of Genetics, Stanford University, Stanford, CA 94305, USA; Joint Initiative for Metrology in Biology, Stanford University, Stanford, CA 94305, USA; SLAC National Accelerator Laboratory, Menlo Park, CA 94025, USA; Department of Genetics, Stanford University, Stanford, CA 94305, USA; Molecular and Computational Biology Section, Department of Biological Sciences, University of Southern California, Los Angeles, CA 90089, USA; Molecular and Computational Biology Section, Department of Biological Sciences, University of Southern California, Los Angeles, CA 90089, USA

**Keywords:** complex traits, host–pathogen, yeast, mice, fungal pathogens, mouse–fungus interactions, mammal–fungus interactions, antagonistic pleiotropy

## Abstract

Determining how genetic polymorphisms enable certain fungi to persist in mammalian hosts can improve understanding of opportunistic fungal pathogenesis, a source of substantial human morbidity and mortality. We examined the genetic basis of fungal persistence in mice using a cross between a clinical isolate and the lab reference strain of the budding yeast *Saccharomyces cerevisiae*. Employing chromosomally encoded DNA barcodes, we tracked the relative abundances of 822 genotyped, haploid segregants in multiple organs over time and performed linkage mapping of their persistence in hosts. Detected loci showed a mix of general and antagonistically pleiotropic effects across organs. General loci showed similar effects across all organs, while antagonistically pleiotropic loci showed contrasting effects in the brain vs the kidneys, liver, and spleen. Persistence in an organ required both generally beneficial alleles and organ-appropriate pleiotropic alleles. This genetic architecture resulted in many segregants persisting in the brain or in nonbrain organs, but few segregants persisting in all organs. These results show complex combinations of genetic polymorphisms collectively cause and constrain fungal persistence in different parts of the mammalian body.

## Introduction

Fungi are a major class of opportunistic human pathogen, infecting billions and killing millions of people per year (Brown *et al.* 2012; Almeida *et al.* 2019). Hundreds of diverse fungal species are known to infect humans (Kohler *et al.* 2014). These fungi mainly infect the immunocompromised, an increasing segment of the global population due to improvements in medicine that have lowered the mortality associated with life-threatening conditions (Kohler *et al.* 2014). Such opportunistic infections can be difficult to treat (Borman *et al.* 2008; Kohler *et al.* 2014; Brown and May 2017; Almeida *et al.* 2019), but the identification of mechanisms enabling fungi to cause these infections may facilitate the development of more effective antifungal therapies (Clemons *et al.* 1994; McCusker *et al.* 1994; Byron *et al.* 1995; Kumamoto 2008a, 2008b; Scorzoni *et al.* 2017; Nivoix *et al.* 2020).

Many opportunistic fungal pathogens can be challenging to work with genetically (Hickman *et al.* 2013; Cavalheiro and Teixeira 2018). However, the budding yeast *Saccharomyces cerevisiae*, one of the main eukaryotic model organisms in biology, is also an opportunistic pathogen, with numerous isolates obtained from clinical infections (Clemons *et al.* 1994; McCusker *et al.* 1994; Byron *et al.* 1995; Goldstein and McCusker 2001; McCusker 2006; Strope *et al.* 2015). Humans are regularly exposed to *S. cerevisiae*, as it occurs naturally in the environment and is used in the production of beer, wine, bread, chocolate, and other foods and dietary supplements (Gallone *et al.* 2016; Ludlow *et al.* 2016; Khatri *et al.* 2017; Peter *et al.* 2018; Fay *et al.* 2019). Notably, *S. cerevisiae* is in the same family of Ascomycete yeasts as *Candida*, the main genus involved in opportunistic fungal infections (Cannon and Kirk 2007).

The ability to infect immunocompromised humans varies among *S. cerevisiae* strains (McCusker *et al.* 1994), with clinical isolates found throughout the species’ genealogy (Liti *et al.* 2009; Strope *et al.* 2015). Despite their lack of genetic relatedness, clinical *S. cerevisiae* isolates are thought to possess similar traits, including the ability to attach to and penetrate surfaces and to tolerate human body temperature (Clemons *et al.* 1994; McCusker *et al.* 1994; Magwene *et al.* 2011; Strope *et al.* 2015; Peter *et al.* 2018; Phadke *et al.* 2018). However, determining why certain strains are able to infect humans ultimately requires mapping the specific genetic polymorphisms that cause opportunistic pathogenicity and determining the traits they affect. Such work is hard because it requires performing genetic mapping in yeast inside mammalian hosts, such as mice.

The most powerful genetic mapping approach in *S. cerevisiae* is linkage mapping with large panels of haploid meiotic progeny (segregants) (Bloom *et al.* 2013; Matsui *et al.* 2022). When 2 haploid isolates are crossed and sporulated, their haploid segregants each receive a unique random combination of alleles from their parents (Ehrenreich *et al.* 2009). This shuffling of genetic material makes it possible to measure traits of interest in segregants and then link these traits to specific genomic locations (loci) that cause phenotypic differences (Rockman 2008). Examination of large numbers of segregants provides the statistical power to identify loci explaining most of the heritable differences in traits of interest (Bloom *et al.* 2013; Matsui *et al.* 2022).

To enable linkage mapping with an *S. cerevisiae* cross in mice, we generated a panel of haploid segregants with known genotypes and chromosomally encoded DNA barcodes (Levy *et al.* 2015; Matsui *et al.* 2022), which enabled high-throughput phenotyping as a pool. We mated the lab reference strain (BY) and a haploid derivative of the 322134S clinical isolate (3S) (Liti *et al.* 2009). BY and 3S are highly diverged at the sequence level, with a genetic difference present every ∼270 bp (Taylor *et al.* 2016; Mullis *et al.* 2018). 3S is an isolate obtained from the throat sputum of a patient with a clinical infection (Liti *et al.* 2009), while BY is a commonly used reference strain that is thought to be avirulent (Clemons *et al.* 1994).

We used the barcoded BY × 3S cross to obtain insights into how genetic differences among strains influence the persistence of yeast in mice. This was done by injecting a pool of 822 barcoded BY × 3S *MAT*ɑ segregants into the mouse bloodstream and enumerating segregant abundances in host organs over time by barcode sequencing. Barcode frequencies provide direct readouts of the frequencies of each of the 822 segregants in an organ at a given time point. These data enabled linkage mapping, which identified numerous loci that explained most of the heritable variation in yeast persistence in hosts. Some loci showed consistent effects across host organs (general loci), while others had counteracting effects across host organs (antagonistically pleiotropic loci; Qian *et al.* 2012; Wei and Zhang 2019; Chen and Zhang 2020), causing different segregants to be superior in different organs. This work advances the use of *S. cerevisiae* as a model for opportunistic fungal pathogenicity and host–microbe interactions.

## Materials and methods

### Generation of haploid segregants

Haploid segregants were generated from a cross of 2 isolates of *S. cerevisiae*, the lab strain BY4716 (BY) and a haploid derivative of the clinical isolate 322134S (3S) (Liti *et al.* 2009). Specifically, BY *ho fcy1Δ flo8Δ flo11Δ ura3Δ YBR209W::pGal1-Cre—*Lox71—Lox2272/71 and 3S *ho fcy1Δ flo8Δ flo11Δ ura3Δ YBR209W::pGal1-Cre—*Lox71—Lox2272/71 parent strains were used. Construction of these strains is described in detail in Matsui *et al.* (2022). In brief, for each of these gene deletions, the coding region was completely removed, without any marker left behind. The *fcy1Δ* and *ura3Δ* gene deletions provide counterselectable markers, while the *flo8Δ* and *flo11Δ* gene deletions should eliminate clumping and flocculation (Liu *et al.* 1996; Lo and Dranginis 1996; Bayly *et al.* 2005), traits problematic for pooling of segregants and recovery of yeast from mouse organs. *FLO8*, *FLO11*, and *FCY1* were previously shown to be neutral with respect to survival of a different *S. cerevisiae* clinical isolate (YJM145) in mouse hosts, while *URA3* is known to negatively impact survival in mouse hosts (Goldstein and McCusker 2001). Also, most yeast natural isolates exhibit cell clumping due to an aspartic acid at the 368th amino acid of *AMN1*, which causes a defect in daughter cell separation during mitosis (Yvert *et al.* 2003; Fang *et al.* 2018). However, lab strains such as BY, as well as ∼0.3% of natural isolates (Peter *et al.* 2018), possess a valine at amino acid 368, which enhances daughter cell separation, preventing clumping. Sequencing of the haploid 3S parent, as well as its diploid progenitor, revealed these strains exclusively carry the same valine, nonclumping allele as lab strains, despite having an *AMN1* that otherwise shares a number of polymorphisms with other wild strains. Thus, clumping was not observed in our cross.

Parental strains were also engineered to have a genomic landing pad (Levy *et al.* 2015; Schlecht *et al.* 2017; Liu *et al.* 2019) with 2 partially crippled LoxP sites (Lee and Saito 1998), Lox71 and Lox2272/71, and a galactose-inducible Cre recombinase (Sauer 1987) at the neutral *YBR209W* locus (Levy *et al.* 2015) (Supplementary Fig. 1a). We first generated *MAT****a*** versions of the BY and 3S parent strains and then obtained *MATɑ* versions through mating-type switching of the *MAT****a*** strains with a galactose-inducible *HO* plasmid (Herskowitz and Jensen 1991). All segregants in a pool should be the same mating type, otherwise mating will occur. To ensure that a pool of segregants of the same mating type could be generated without any allelic bias near the mating locus, we created 2 BY/3S heterozygous diploids: BY *MAT****a*** × 3S *MATɑ* and 3S *MAT****a*** × BY *MATɑ.* Both diploids were sporulated and roughly equal numbers of 4-spore tetrads (∼500) were obtained from each by tetrad dissection. To maximize the number of unique recombination breakpoints in our panel of segregants, 1 *MATα* segregant was then randomly selected from each tetrad for inclusion in this study.

### Barcoding of haploid segregants

A total of 822 segregants were barcoded through the transformation and integration of a barcoded plasmid library via Cre-mediated homologous recombination at Lox2272/71 (Supplementary Fig. 1). A pBAR6 plasmid (Liu *et al.* 2019) marked with *KanMX* was modified by Gibson assembly to include a Lox2272/66 site, a random 20-mer barcode sequence, and a partial TruSeq read 2 adapter sequence. The barcoded plasmids were transformed into each segregant individually and then recombined into the yeast genome by inducing the galactose-inducible Cre recombinase using YP + 2% galactose media for ∼20 h. Cre-mediated recombination between the 2 partially crippled Lox2272 variants, Lox2272/66 and Lox2272/71, produces a crippled Lox2272/66/71 and a fully functional Lox2272. For each segregant, all integrants (between 1 and 5) were picked from YPD + 200 µg/ml G418 agar plate. Glycerol freezer stocks of these integrants were then made and stored at −80°C. A subset of 86 segregants containing 3 different barcodes were included in all work, enabling internal replication and measurement of broad-sense heritability within samples.

### Whole-genome sequencing of haploid segregants

Genomic DNA was obtained from each segregant using the Qiagen DNeasy Blood and Tissue kit. For each segregant, a whole genome sequencing library was then constructed using the Illumina Nextera kit. Each library was barcoded and ∼192 segregants were multiplexed per sequencing lane. Sequencing libraries from segregants were pooled in equimolar fractions, size selected from an agarose gel, and purified using the Qiagen Gel Extraction kit. Multiplexed samples were sequenced by Novogene on 6 Illumina HiSeq 2500 lanes using 150 bp × 150 bp paired-end reads. Reads for each segregant were mapped against the S288c reference genome R64-2-1_20150113 using BWA with default settings (Li and Durbin 2009). Using SAMTOOLS (Li *et al.* 2009) with default settings, alignments were converted to bam files and sorted, read duplicates were removed, and pileups were generated. Data for 43,865 high-confidence SNPs (Mullis *et al.* 2018; Matsui *et al.* 2022) that differ between BY and 3S was then extracted from the pileup files. Segregants showing any signs of aneuploidy or cross-contamination in their genotype data were excluded from further analysis. Also, all segregants with a mean per site coverage of less than 2 were removed. In total, 76 *MATα* segregants were removed based on these criteria. For the remaining 927 *MATα* segregants, a vector containing the fraction of 3S calls at each SNP was generated. Initial genotype calls were made by classifying sites above and below 50% classified as 3S and BY, respectively. A hidden Markov model (HMM) was then used to correct these initial genotype calls and impute information at missing sites. The HMM was implemented using the HMM package version 1.0 (Himmelmann 2015) in R. We employed the transition and emission probability matrices: transProbs = matrix(c(0.9999, 0.0001, 0.0001, 0.9999)) and emissionProbs = matrix(c(0.25, 0.75, 0.75, 0.25)). Adjacent SNPs in the HMM-corrected genotype calls that lacked recombination in the segregants were collapsed, as they contained identical information. This reduced the number of SNP markers in subsequent analyses from 43,865 to 14,347.

### Determination of segregant barcodes

To determine the barcode(s) in each segregant, we performed targeted Illumina sequencing. Libraries were generated via PCR using custom primers flanking the barcode. The primers used to amplify the barcode were:

Forward:

5′-**TCGTCGGCAGCGTCAGATGTGTATAAGAGACAG**ACGAAGTTATTGCGCGGTGATC -3′

Reverse:

5′-**GTCTCGTGGGCTCGGAGATGTGTATAAGAGACAG**GGTACGTGTGCTCTTCCGATCT -3′,

where the sequences in bold are TruSeq adapter sequences and the remainder of the sequence is homologous to the flanking sequence in the genomic landing pad. PCR products were purified using a Qiagen MinElute PCR purification kit, amplified using barcoded TruSeq adapters and Illumina P1 and P2 primers, and then size selected via gel extraction prior to sequencing. Sequencing was performed using Novogene Illumina HiSeq 2500 150 bp × 150 bp paired-end reads at ∼2,000× coverage per barcode. 20-mer barcode sequences for each segregant were extracted from the sequencing reads and clustered with Bartender (Zhao *et al.* 2018) using a Hamming distance of 2. Clusters comprising >5% of the total reads for each sample were considered true barcodes. Only *MATα* segregants that passed genotyping quality thresholds and had at least 1 barcode different from all others were used in this study.

### Animal husbandry

All procedures and personnel involving mice were approved by the University of Southern California’s Institute for Animal Care and Use Committee under protocol #21102. All mice were housed on a 14:10 h light: dark cycle and had ad libitum access to food and water. A single mouse strain, C57BL/6J (JAX stock #000664), of *Mus musculus* was used for all experiments. Mice were ordered from the Jackson Laboratory at 6 weeks of age and housed for 2 weeks prior to experiments. Upon arrival, mice were housed in groups of 3 according to their sex, then housed individually beginning at 7 weeks of age until they were euthanized at their experimental endpoints (1, 2, or 5 days postinfection). To minimize batch effects, animals in the same cage were parsed into different combinations of dexamethasone treatment or time point.

### Experimental infection of mice

A total of 36 mice (2 sexes × 2 dexamethasone treatments × 3 time points × 3 replicates) were used in the following experiments. At 8 weeks of age, individuals were split into immunocompromised (dexamethasone+) or immunocompetent (dexamethasone-) treatments. To generate immunocompromised animals, 500 µl of 4 mg/ml dexamethasone sodium phosphate (Sigma-Aldrich) was administered twice daily at ∼9 AM and ∼9 PM to animals via intraperitoneal (IP) injection. Immunocompetent (dexamethasone-) control animals were treated with 500 µl water twice daily via IP injection. These treatments began 2 days prior to infection and continued until experimental endpoints. Daily treatments were administered at regular intervals, with the first injection administered between 9 and 10 AM and the second between 9 and 10 PM. To prevent infection by agents other than the panel of haploid segregants, we also gave dexamethasone-treated animals water with 0.1 mg/ml gentamicin sulfate and 2 mg/ml streptomycin sulfate. Animals injected with water were provided water without antibiotics.

Two days prior to infection, the yeast strains used in this study were grown from frozen stocks to stationary phase in 800 µl YPD (∼2 days) in 96 well plates. Strains were resuspended and equal volumes (100 µl) of each strain were pooled and mixed to ensure homogeneity. Cells from the pool were harvested, washed with water, and resuspended in 10 ml of water. Cell concentration was calculated using a hemocytometer and 1 × 10^7^ cells in 100 µl water from the pool were used to inoculate the mice via tail vein injection. Prior to the injection, the tails of the mice were dipped in warm water to help dilate the tail vein. Because the C57BL/6J are dark-furred mice, a Leica KL 500 LCD light source was used to visualize the tail vein more clearly during injection. C.G. and M.L.S. performed all injections. To minimize batch effects, injection order was randomized with respect to dexamethasone treatment and time point, with CG injecting all “odd” mice and MLS. injecting all “even” mice. Prior to injection, we took a sample of the pool of segregants to estimate initial frequencies.

### Organ harvesting and processing

At each experimental endpoint (1, 2, or 5 days postinfection), animals were euthanized via cervical dislocation and wiped down with 70% EtOH to disinfect them prior to dissection. From each of the 36 mice, 5 distinct organs were dissected and removed from each animal in the following order: liver (only the 2 largest lobes by weight were used), right and left kidney, spleen, gonads, and brain, for a total of 180 samples. Samples were split and transferred into 2 Qiagen PowerBead Tubes (Metal 2.38 mm) each (1 lobe of liver per tube, 1 kidney per tube, half of the brain, gonad, and spleen samples per tube) and 1 ml of 1× TrypLE Select Enzyme (Thermo Fisher 12563029) was added to each tube. Organ samples were incubated at 37°C for 5 min and then homogenized at 30 Hz for 3 min using a Qiagen Tissuelyzer II. Homogenized samples were plated on YPD agar plates containing 200 µg/ml G418 and grown for 2 days at 30°C to allow yeast colony formation. About 1:50 and 1:100 dilutions of each organ sample were also plated to ensure accurate colony counts could be taken in case of crowding on the plates. After 2 days of growth, plates were imaged using a BioRad Gel Doc XR+ with white light and an exposure time of 0.5 s. Next, 10 ml of water was added to the surface of each plate, and colonies were scraped off of the surface. Resuspended yeast samples were vortexed to ensure homogeneity, harvested via centrifugation, and stored at −20°C prior to sequencing.

### YPD plate growth controls

After pooling strains for injection, aliquots of cells were subjected to the tissue homogenization process prior to plating on YPD agar plates containing 200 µg/ml G418. This process involved harvesting cells and resuspending them in 2 ml of 1× TrypleSelect enzyme, incubating the cells at 37°C for 5 min in Qiagen PowerBead Tubes (metal 2.38 mm), and homogenization at 30 Hz for 5 min using a Qiagen Tissuelyser II. Cells were plated at densities of 10^4^, 10^5^, 10^6^, and 10^7^ cells per plate in triplicate. Control plates were grown at 30°C for 2 days before imaging and collecting cells as described above. Dilutions of each plated sample were also plated to assess the number of colonies formed per plate. After collecting yeast from the plates, samples were vortexed to ensure homogeneity, harvested via centrifugation, and stored at −20°C prior to sequencing. To measure the effect of the tissue homogenization process on yeast cells, separate aliquots of cells were plated directly onto YPD agar plates containing 200 µg/ml G418 at 10^4^,10^5^, and 10^6^ cells per plate in duplicate without being subjected to tissue homogenization. These untreated controls were grown, imaged, harvested, and stored in the same manner as those described earlier.

### Barcode library preparation

Frozen cultures were thawed and DNA was extracted using the Zymo Quick-DNA Fungal/Bacterial Minprep Kit. Quantification of DNA was performed using a Qubit high-sensitivity assay. After DNA extraction, a 2-step PCR was used to amplify the barcoded region of the genome for sequencing. We amplified 150 ng of DNA per organ sample or control, corresponding to ∼1.16 × 10^7^ genomes or ∼11,670 copies per barcode (994 barcodes total; 736 strains barcoded once and 86 strains barcoded in triplicate). We performed a 4-cycle PCR on each sample using Phusion polymerase, 150 ng of DNA, and 1 µl of 10 µM primers each at a total reaction volume of 50 µl. The primers used in this reaction were:

Forward:

5′-AATGATACGGCGACCACCGAGATCTACAC**NNXXXXNN**ACACTCTTTCCCTACACGACGCTCTTCCGATCTACGAAGTTATTGCGCGGTGA-3′

Reverse:

5′-CAAGCAGAAGACGGCATACGAGAT**NNXXXXNN**GTGACTGGAGTTCAGACGTGTGCTCTTCCGATCT-3′,

with Ns in this sequence corresponding to random nucleotides at a frequency of A: 25%, C: 25%, G: 25%, T: 25%. These random sequences were used as unique molecular identifiers (UMIs), enabling identification of PCR duplicates in downstream analyses. Xs in the above sequences correspond to known, custom multiplex tags, which are used to demultiplex reads from different samples when pooled onto the same flow cell for sequencing. Multiplexing tags were designed to have a Hamming distance of 4 from one another. The 4-cycle PCR reaction was performed using the following steps:

1. 98C, 3:00 min
2. 98C, 0:30 s
3. 54C, 0:30 s
4. 72C, 2:00 min
5. Repeat steps 2 through 4 4×
6. 72C, 5:00 min
7. 4C, hold indefinitely

After amplification, samples were purified using MinElute 96 UF PCR Purification Kit (Qiagen) protocol and eluted into 20 µl of water. Next, a 24-cycle PCR was performed using 15 µl of purified library and Phusion Polymerase (New England Biolabs) in a total reaction volume of 50 µl. TruSeq F and R primers were used at 10 µM concentration for this PCR. The 24-cycle PCR was performed using the following steps:

1. 98C, 3:00 min
2. 98C, 0:30 s
3. 54C, 0:30 s
4. 72C, 2:00 min
5. Repeat steps 2 through 4 24×
6. 72C, 5:00 min
7. 4C, hold indefinitely

A total of ∼45 µl of each PCR product were pooled together and the appropriate PCR product (∼220 bp) was isolated by gel electrophoresis and extracted using a QIAquick Gel Extraction Kit. The pooled samples were checked for purity using a Nanodrop ND-1000 and concentration using a Qubit Fluorometer and shipped to Novogene. Prior to sequencing, the pooled libraries were further quantified by Agilent Bioanalyzer at Novogene.

### Barcode sequencing

Samples were sequenced on an Illumina HiSeq 4000 using a single flow cell. Because the majority of the nucleotides in the barcode amplicons are fixed, a 25% PhiX DNA spike-in was done to increase read diversity. Sequencing reads were analyzed using custom scripts in R and Python. Reads were demultiplexed and discarded if the average Illumina quality score of the read in the barcoded region was less than 30 or the landing pad sequence AGTATCCTATACGAACGGTA adjacent to the barcode was not present in the read. PCR duplicates were eliminated by excluding reads within samples that contained the same UMI sequences (only 1 copy was retained). In each file, barcodes were clustered using Bartender (Zhao *et al.* 2018) using a maximum allowable clustering distance of 3 (bartender_single_com -f *$infiles* -o ${infiles%.*} -d *3*). Raw counts were obtained for each present barcode and values were normalized to the total number of barcode reads in the sample.

### Barcode quantification and phenotype calculation

For each segregant in each sample, we computed the persistence phenotype as (*f*_TF_ − *f*_T0_)/*f*_T0_, with *f*_T0_ and *f*_TF_ corresponding to a segregant’s barcode frequency in the initial pool and a sample, respectively. These raw phenotypes were calculated for both control and organ samples. We accounted for potential impacts of outgrowing samples on YPD plates using the fixed effects linear model *phenotype_sample_ ∼ phenotype_control_* + *error*. In this model, *phenotype_sample_* and *phenotype_control_* corresponded to individuals’ raw phenotypes in organ samples and mean raw phenotypes in controls, respectively. This model was implemented using the lm() function in R. The mean raw phenotype of controls was calculated using 3 YPD control samples plated at 10^5^ cells per plate. We refer to the residuals extracted from this model as “persistence”—for example, genotypes with large residuals were at higher frequency compared to controls. For the 86 segregants that were replicated in the pool with distinct barcodes, all replicates were separately corrected. These replicates were only utilized in heritability analyses. In all work with the full set of 822 segregants, only a single barcode replicate was utilized for each replicated segregant. Because of logistical considerations, initial pool, control, and samples had to be collected at different time points.

### Identification of samples with significant variation among segregants

To identify samples in which segregants showed significant phenotypic differences, we performed 1-way analysis of variance (ANOVA) analysis. For each sample, we conducted a test using the fixed effects linear model *phenotype ∼ genotype* + *error*, which was implemented with the lm() function in R. The *P*-value of each model was recorded and significant samples were identified using a conservative Bonferroni-corrected threshold (ɑ = 0.05/139, samples = 139). Nonsignificant samples were excluded from further analysis.

### Calculation of broad-sense heritability within and across samples

Within each sample, broad-sense heritability (*H*^2^) was calculated using the 86 segregants that were replicated in the segregant pool. We used a mixed effects linear model with the formula *phenotype ∼ genotype* + *error*, with *genotype* a categorical, random effect variable corresponding to the identities of replicated segregants. This model was implemented using the lmer() function in the lmer package in R (Bloom *et al.* 2013; Matsui *et al.* 2022). Within-sample *H*^2^ was computed by taking the sum of squares of *genotype* and dividing it by the total sum of squares of the model.

We also calculated *H*^2^ across all or subsets of samples using the persistence data of all 822 segregants, including only the first replicate for the 86 replicated segregants. Across-sample *H*^2^ was calculated as described above for within-sample *H*^2^.

### Linkage mapping in individual samples

Linkage mapping was performed on each individual organ sample using forward regression. A total of 14,347 SNPs distributed throughout the genome were employed as markers, with each categorically encoded as “0” or “1” for the BY or 3S alleles, respectively. In first stage scans, the fixed effects linear model *phenotype ∼ locus* + *error* was implemented for each marker in a sample using the lm() function in R. In these models, *phenotype* corresponded to raw phenotypes and persistence measurements in control and organ samples, respectively, and *locus* corresponded to individuals’ genotypes at a marker. For each test, the *P*-value of the *locus* term was extracted using the R function summary.aov(). Significance thresholds were determined separately for each sample using 1,000 permutations (Churchill and Doerge 1994) of the segregant persistence data. In each permutation, the *phenotype* vector was randomly shuffled while the matrix of genotypes was held constant. A genome-wide scan was performed on each permuted dataset using the fixed effects linear model *phenotype ∼ locus* + *error*, with the minimum *P*-value in each permutation recorded. The threshold for a first stage scan in a sample was determined based on the fifth percentile of its minimum *P*-values from permutations. We only allowed detection of a single locus per chromosome per scan. Conservative confidence intervals (95%) were determined for each locus using 2 × −log_10_(*P*-value) drops from a peak marker at a locus.

After the first stage scans, additional scans were performed on each sample. In these scans, we used the fixed effects linear model *phenotype ∼ known_locus_1_ + … known_locus_N_ + locus* + *error*, with each *known_locus* term corresponding to individuals’ genotypes at loci identified in earlier scans. The *phenotype* and *locus* terms were defined in the same manner as in the first stage scans. In each additional scan, permutations were conducted in the same manner as the first stage scans, except with *known_locus* terms also included. In these additional scans, we allowed detection of only a single locus per chromosome per scan and confidence intervals were computed in the same way as the first stage scan. Scans were continued until no loci were detectable at permutation-based significance thresholds.

### Consolidation of loci detected in individual samples

Loci detected in individual organ samples were consolidated across samples according to their 2 × −log_10_(*P*-value) confidence intervals. Two loci were considered the same if these intervals overlapped. The intersection and union of all consolidated confidence intervals were recorded; the intersection of all confidence intervals was used to resolve loci (Supplementary Table 2), while the union was used to determine which detections were consolidated into each locus (Supplementary Data 8).

### Aggregation of persistence measurements across samples

Brain and nonbrain samples with statistically significant differences in persistence were identified. Segregant phenotypes (raw measurements) were first time corrected by dividing by the number of days since injection into a host. Next, a fixed effects linear model was fit to account for growth on rich medium as described above. Each segregant’s residual measurements (persistence) were then averaged across all 15 brain or 75 nonbrain samples. Differential persistence between brain and nonbrain samples was computed by taking the difference between a segregant’s mean brain and mean nonbrain time-corrected persistence values.

### Linkage mapping with aggregate phenotype data

We performed linkage mapping with aggregate brain and nonbrain persistence measurements, as well as the difference between the 2. All aspects of these 3 forward regression scans were implemented in the same manner as the linkage mapping scans on individual samples. The only difference was that in these scans with aggregate data, *phenotype* in these models was the aggregate brain data, the aggregate nonbrain data, or the difference between the 2.

### Calculating the effects of loci in individual samples

For each consolidated locus, the effect size was calculated in each of the individual samples with significant heritabilities as well as in the control samples. Effect sizes in the controls were calculated using the raw phenotypes while effect sizes in the organ samples were calculated using the residual values after correcting for segregant growth on YPD (described in the “Barcode Quantification and Phenotype Calculation” section). To calculate the effect size of a locus in a particular organ or control, phenotype data for the sample was split by genotype at the locus. Mean phenotypes were calculated for individuals with the BY and 3S alleles and the difference between these means (3S−BY) was computed. Effect sizes were only calculated in organ samples with significant phenotypic variation.

### The effects of individual loci identified in aggregate phenotype data

We computed the effects of the 18 loci detected in the linkage mapping scans with aggregate data using both the aggregate brain and aggregate nonbrain persistence measurements. For every locus, we subtracted the mean aggregate brain measurement among segregants with the BY allele from the mean aggregate brain measurement among segregants with the 3S allele. The same procedure was then repeated using the aggregate nonbrain measurements. A positive value in the brain or nonbrain organs implies the 3S allele was beneficial and the BY allele was detrimental, while negative value indicates the opposite relationship. About 95% bootstrap confidence intervals were computed for these values by the sampling phenotypes of individuals with BY or 3S alleles at a given locus 1,000 times with replacement using the sample()function in R with replace = T, computing the difference in mean brain or nonbrain phenotype between these 2 groups for each sampling, and taking the 2.5th and 97.5th percentiles from these differences. Loci with positive or negative values in both brain and nonbrain organs were classified as having general effects. By contrast, loci showing counteracting effects between brain and nonbrain organs were categorized as antagonistically pleiotropic.

### Combinatorial effects of loci identified in aggregate phenotype data

We first determined the alleles present in each segregant at the 18 loci detected in the aggregate scans. The effect size at each locus was calculated in both brain and nonbrain samples by subtracting the mean persistence of segregants containing the BY allele from the mean persistence of segregants containing a 3S allele at a given locus. Segregants were considered “enriched” for generally beneficial alleles if they contained 7 or more generally beneficial alleles and “depleted” if they contained fewer than 3. At pleiotropic loci, segregants were considered “enriched” for brain loci if they contained 6 or more loci favoring persistence in the brain over nonbrain organs, and “depleted” if they contained fewer than 3. These thresholds were established so to include enough segregants to enable contingency tests on segregants were “enriched” or “depleted” for both general and pleiotropic loci to test the combinatorial effects of the loci; however, no more than 25% of segregants in the dataset were considered “enriched” or “depleted” for generally beneficial or pleiotropic loci.

Fixed-effect linear models were employed to test the relationship between mean segregant persistence in brain or nonbrain samples and the number of generally beneficial or pleiotropic loci present in each segregant. These models were implemented using the lm() function in R and took the form *phenotype ∼ num_loci + error*, where *phenotype* corresponds to segregants’ mean persistence values in either the brain or the nonbrain samples, and *num_loci* corresponds to the number of either generally beneficial alleles or the number of pleiotropic alleles favoring persistence in the brain. *P*-values were extracted from these models using the summary.aov() function and *R*^2^ values were extracted using the summary() function.

Contingency tests were performed to determine whether segregants with increasing persistence measurements over time in brain or nonbrain samples were enriched for generally beneficial alleles as well as pleiotropic alleles favoring persistence in a particular sample type. To do this, the linear model *phenotype ∼ time* + *error* was fit for each segregant using the lm() function in *R*, where *phenotype* was a segregant’s phenotype in each of the 15 brain or 76 nonbrain samples, and *time* was a numeric vector encoding the number of days postinjection that each sample was collected. The *time* coefficient from each model was extracted using the coefficients() function and used in a 2 × 2 contingency test in which segregants were grouped by whether they were enriched for both beneficial general and pleiotropic alleles or not as well as whether they had a positive coefficient or not.

To visualize the collective effects of general and pleiotropic loci on strain persistence over time, mean time point 1 and time point 5 phenotypes for all strains were calculated separately for brain and nonbrain samples. These mean phenotypes were then normalized to the time point 1 phenotypes (by subtracting the time point 1 phenotype). Strains were divided into 4 focal classes depending on whether they had ≥X or ≤X beneficial alleles at general loci and ≥X or ≤X alleles associated with brain persistence at pleiotropic loci. Within these 4 groups, the mean normalized time point 5 phenotype and standard error were calculated. Bootstrapping was also performed using 1,000 samplings of the mean time point 5 persistence values within each focal group. The slope of the mean segregant persistence between the beginning (day 1) and end (day 5) of the experiment were then plotted for each focal group, along with the slopes resulting from the 1,000 bootstrap samplings (Fig. 5, c and d).

### Genes underlying loci detected in aggregate scans

To resolve the loci identified using aggregate scans, we utilized loci detected in the individual organ samples. For each consolidated aggregate locus, we first determined the smallest bounds for the locus among the 3 scans using aggregate persistence measurements. Next, we determined which loci from individual samples had 95% confidence intervals overlapping these bounds. After all individual loci had been identified, we utilized these confidence intervals to find the most narrow windows for each locus. Genes within each window were identified based on the R64-2-1_20150113 *S. cerevisiae* genome annotation from the *Saccharomyces* Genome Database (Cherry *et al.* 2012).

## Results

### Generation and barcoding of haploid segregants

We crossed haploid BY and 3S strains that were genetically engineered to produce segregants amenable to pooled, high-throughput phenotyping. *FLO11* and *FLO8*, which respectively encode the main cell surface flocculin in this organism (Lo and Dranginis 1996; Bayly *et al.* 2005) and its primary transcriptional activator (Liu *et al.* 1996), were deleted from these strains prior to mating. These deletions eliminate cell clumping and flocculation within and between segregants, which are problematic for pooled experiments. However, they also diminish surface adhesion and invasion, limiting our insight into these traits. BY and 3S were also engineered to have a genomic landing pad at the *YBR209W* locus, enabling site-specific integration of barcodes into segregants (Levy *et al.* 2015; Liu *et al.* 2019; Matsui *et al.* 2022). These engineered BY and 3S strains were mated to produce a BY/3S diploid, which was sporulated.

To ensure balanced allele frequencies and random multilocus genotypes among segregants, we performed tetrad dissection and randomly chose and barcoded 1 *MAT*α haploid from each of 822 tetrads using transformation with a random barcode library (Levy *et al.* 2015; Liu *et al.* 2019; Matsui *et al.* 2022) (Supplementary Fig. 1 and Supplementary Data 1). A total of 86 segregants were marked with 2 additional random barcodes and these replicates were also included in our experiments. These internally replicated segregants enable estimation of heritability within samples, while limiting the total number of unique barcodes. Pilot experiments suggested the number of yeast that could be recovered from mouse organs varied by orders of magnitude, with the lowest recoveries in the hundreds and low thousands, constraining the total number of barcodes that could be used in the pool.

Illumina sequencing was used to genotype each of the 822 segregants and determine their barcode(s). All 822 barcoded segregants and their replicates were then grown individually to stationary phase and combined into a single pool in equimolar fractions. All experiments reported in this manuscript involve using barcode sequencing to track changes in segregant frequencies relative to this initial pool.

### Injection of yeast into mice and recovery of yeast at 3 time points

**Fig. 1.**
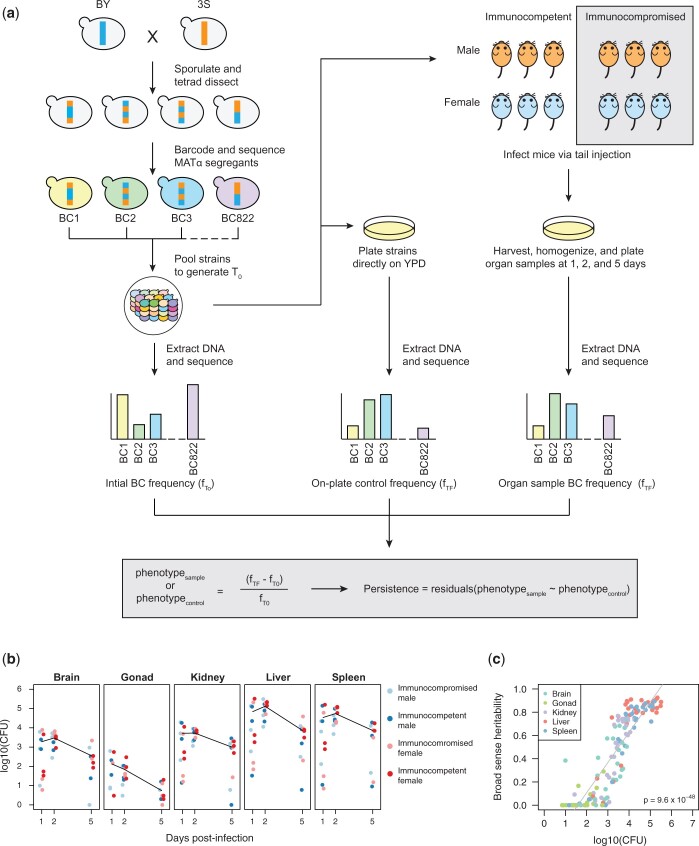
Experimental infections of mice using a pool of barcoded, haploid segregants. a) The workflow and general design of the experiment. Haploid BY and 3S strains were crossed, the resulting diploid was sporulated, and tetrads were dissected to generate a panel of 822 recombinant haploid progeny. These strains were then barcoded with unique 20-mer nucleotide sequences (barcodes) and pooled together (*T*_0_). The initial pool generated at *T*_0_ was used to infect mice by tail vein injection and simultaneously plated in triplicate onto control plates containing rich medium. At 3 time points postinfection, organ samples were also collected from infected mice and plated. After 2 days of growth, DNA was extracted from the yeast and sequenced to measure relative barcode abundance of each strain. DNA was also extracted from the *T*_0_ sample to measure barcode frequencies in the initial pool. For each strain, the change in normalized barcode frequency relative to *T*_0_ after correcting for on-plate growth was used to calculate persistence. b) CFU recovered from each organ sample across time points. Each panel shows the samples from a different organ. Color of each point corresponds to the sex and immunological state of the mouse from which the sample was recovered. Mean log_10_(CFU) over time is shown as a black line. c) Broad-sense heritability (*H*^2^) of each organ sample as a function of the CFU recovered from that sample. The color of each sample corresponds to organ type.

Thirty-six mice were infected with 1 × 10^7^ cells from the segregant pool by tail vein injection (Fig. 1a). Equal numbers of male, female, immunocompromised (injected with 500 μl of 4 mg/ml dexamethasone [500 μl]), and immunocompetent (injected with 500 μl of water) mice were included. No morbidity or mortality was observed through the entire experiment. One-third of the mice were euthanized at each of 3 time points (1, 2, and 5 days postinjection). From each mouse, we dissected the brain, gonads, kidneys, liver, and spleen. Each organ was homogenized and plated on selective media to isolate yeast from the mouse cells. On average, 69,150 colony forming units (CFU) were recovered per liver, 32,032 CFU per spleen, 3,843 CFU per kidney pair, 1,741 CFU per brain, and 69 CFU per gonad pair (Supplementary Data 2). For every organ, recovery decreased over time, suggesting clearance of at least some segregants by the mice (Fig. 1b). Recovery was lowest in the brain and gonads, which both have blood barriers (Daneman and Prat 2015; Mruk and Cheng 2015; Profaci *et al.* 2020).

### Barcode sequencing and estimation of persistence across yeast genotypes

We define persistence, the focal phenotype of this article, as the tendency for a segregant to remain within a host organ over time as estimated from barcode sequencing. Because sampling was terminal for mice, we could not repeatedly sample yeast from host organs. Thus, we instead calculated persistence in a given organ sample as the fractional change in a segregant’s barcode frequency relative to the initial pool, corrected for the plating step required to recover yeast from mouse organs. Specifically, we measured each segregant’s change in frequency in a given sample relative to its frequency in the initial pool using the formula (*f*_TF_ − *f*_T0_)/*f*_T0_, with *f*_T0_ and *f*_TF_ corresponding to a segregant’s barcode frequency in the initial pool and a sample, respectively (Fig. 1a and Supplementary Data 3). We then used a linear model to correct these changes over time for differences among segregants in on-plate growth (Supplementary Fig. 2 and Supplementary Data 4 and 5).

Of 180 processed samples (5 organs × 2 sexes × 2 dexamethasone treatments × 3 time points × 3 replicates), yeast were recovered from 139. We calculated across-sample broad-sense heritability (*H*^2^) using these 139 samples. In this across-sample heritability analysis, all segregants in a sample were employed, with only a single barcode included for the 86 internally replicated segregants. When we included all samples, the across-sample *H*^2^ was 0.01. Among these 139 samples were 29 brain, 18 gonad, 30 kidney, 31 liver, and 31 spleen samples; we also computed across-sample heritability separately within each of these organs. The brain, gonad, kidney, liver, and spleen samples showed across-sample *H*^2^ values of 0.007, 0.004, 0.072, 0.248, and 0.105, respectively. Collectively, these findings imply that the data are quite noisy and genetic factors explain little of the phenotypic variance across samples when all samples are included.

### Persistence of yeast in mice is highly heritable

To better enable utilization of the data, we identified samples in which segregants showed significant differences in persistence. For each of the 139 samples, we used the 86 segregants that were replicated in the pool to estimate within-sample *H*^2^ in each sample (Supplementary Data 6). In this analysis, *H*^2^ ranged from 0 to 0.92 (median *H*^2^ = 0.57). Our ability to measure within-sample *H*^2^ was strongly affected by yeast recovery from samples, with higher recovery resulting in higher within-sample *H*^2^ (simple linear regression of within-sample *H*^2^ on CFU, *R*^2^ = 0.77, *P* = 9.6 × 10^−48^; Fig. 1c). Variability in yeast recovery among samples was presumably due to both differences in clearance among mice and organs, as well as technical factors associated with organ dissociation. Despite limitations associated with recovering yeast from dissociated organs, the high within-sample *H*^2^ values in many samples shows that genetic polymorphisms among segregants caused differences in persistence in hosts.

To distinguish samples with significant heritable differences in persistence, we applied 1-way ANOVA to the replicated segregants in each sample. A total of 94 samples were statistically significant (Bonferroni-corrected ɑ = 0.05 threshold, *P* ≤ 3.7 × 10^−4^; Supplementary Data 6), indicating significant within-sample *H*^2^. Of these, 2 were excluded because they had distorted measurements for persistent segregants, suggesting their sequencing libraries were of low quality (Supplementary Fig. 3). Only a single gonad sample showed significant differences in persistence among segregants; this sample, which had a relatively low within-sample *H*^2^ value (0.29), was also omitted from later analyses due to a lack of organ replicates (Supplementary Fig. 3). In the 91 remaining significant samples from the brain, kidneys, liver, and spleen, the median within-sample *H*^2^ was 0.75 (from 0.24 to 0.92), indicating most of the variability among segregants in these replicated, higher quality samples was genetic in origin (Fig. 1c).

Supporting a large genetic component to persistence, we again calculated across-sample *H*^2^, here only including the subset of 91 samples. When we did this, the across-sample *H*^2^ was 0.37, and the brain, kidney, liver, and spleen samples showed across-sample *H*^2^ values of 0.35, 0.46, 0.55, and 0.63, respectively. Thus, subsetting the samples based on within-sample *H*^2^ substantially increased across-sample *H*^2^ values and suggested that a substantial part of the genetic basis of persistence is reproducible across samples.

### Segregants exhibit different persistence in the brain than the kidneys, liver, and spleen

**Fig. 2.**
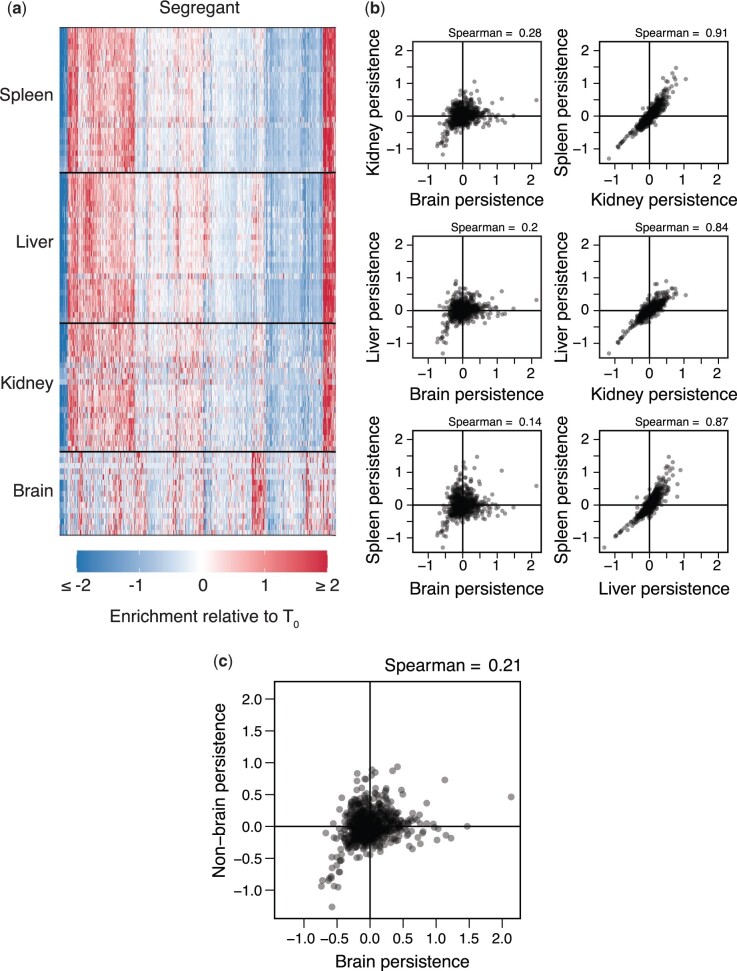
Organ type is the main driver of variation in persistence across samples. a) Heatmap showing persistence of strains (x-axis) across organ samples (y-axis). Samples are clustered by organ type and segregants. b) All pairwise comparisons of segregant persistence phenotypes between organs. Here, each segregant phenotype in an organ represents its average measurement across all samples with heritable differences in persistence. c) Comparison of segregants’ aggregate phenotypes in the brain and nonbrain samples.

We combined the brain, kidney, liver, and spleen samples with significant within-sample *H*^2^ values into a 91 (samples) × 822 (segregants) matrix, with each element corresponding to the persistence of a given segregant in particular sample. We then analyzed the relationships among the 91 samples using hierarchical clustering, principal components analysis (PCA), and examination of pairwise correlations. All methods found the same result: the samples split into 2 clusters, brain and nonbrain (kidneys, liver, and spleen) (Fig. 2a and b and Supplementary Fig. 4). Persistence differences were reproducible across samples in each cluster despite lower recovery of yeast from brain and kidney samples. In PCA, the 2 groups were visible in the loadings on the first principal component (PC_1_), which was the only PC to account for a meaningful portion of the variance across samples (54.1%; other PCs explained ≤7.4% of the variance across samples; Supplementary Table 1). Whether a sample was from the brain or a nonbrain organ was the only experimental factor showing a major relationship with PC_1_, explaining 85.2% of the variance in PC_1_ in a 1-way ANOVA (*P* = 8.84 × 10^−39^). Time and the interaction between brain versus nonbrain and time also had minor significant effects, each explaining <2.7% of the variance in PC_1_ (full-factorial ANOVA with brain vs nonbrain, time, and brain vs nonbrain–time interaction, factor effect test *P* < 2.94 × 10^−3^). Such time effects would be expected if selection acts on phenotypic differences among segregants and these differences vary across organs. Immunological state and sex showed no relationship with PC_1_, perhaps because we directly injected yeast into the bloodstream.

Following these results, we generated aggregate brain and nonbrain persistence measurements for each segregant by averaging data from the 15 brain samples and 76 nonbrain-samples, respectively. As mentioned above, the across-sample *H*^2^ of persistence in the brain was 0.35. Calculation of the across-sample *H*^2^ for nonbrain persistence found a value of 0.52. The aggregate brain and nonbrain measurements showed a poor but highly significant correlation (Spearman’s rho = 0.21, *P* = 1.78 × 10^−9^; Fig. 2c). This suggests that a combination of shared and distinct genetic factors enable persistence in different parts of the host body.

### Genetic mapping of persistence in individual organ samples provides limited power

**Fig. 3.**
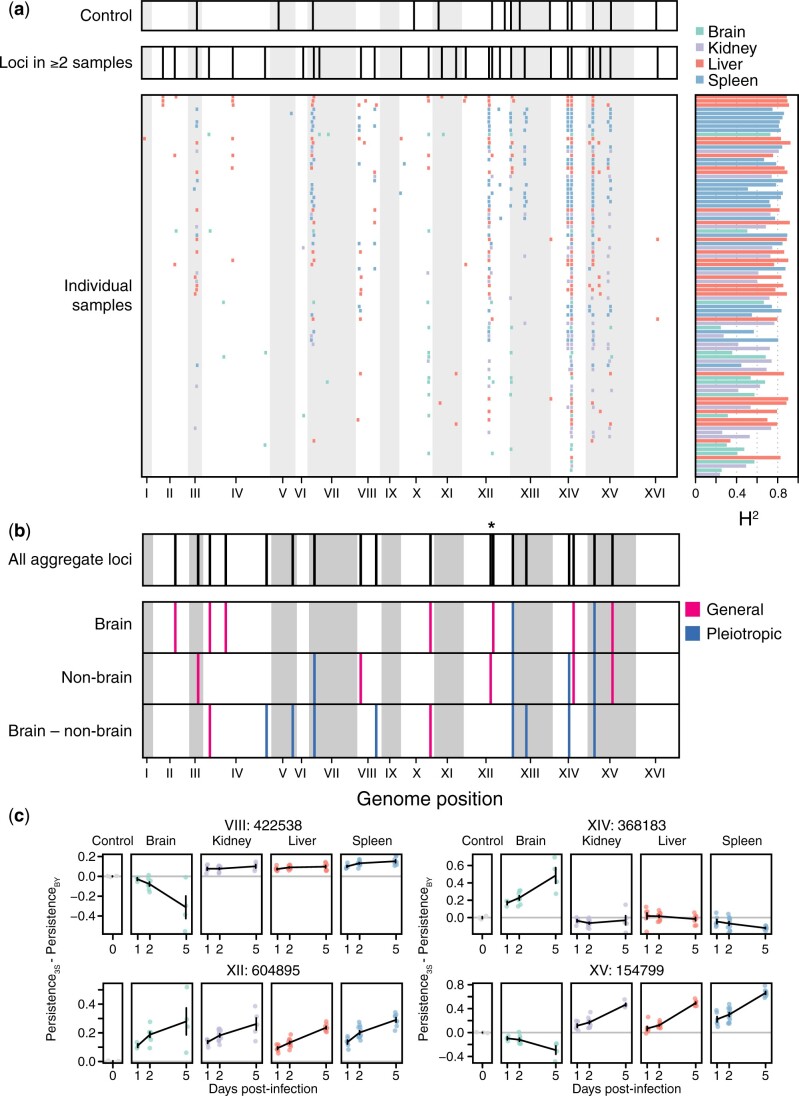
Identification of loci associated with persistence in the host in organ samples. a) Consolidated loci in the plate controls (*top*), consolidated loci across all organ samples (*middle*), and individual loci detected in each organ sample (*bottom*) shown in descending order from greatest to least number of loci detected. Corresponding broad-sense heritability (*H*^2^) measurements for each sample are shown to the right of each individual sample. Samples are colored by organ type. b) Loci detected in genome-wide scans using aggregate data across samples (*top*), followed by loci detected using mean segregant phenotypes in brain and nonbrain samples, as well as the difference in mean phenotype between brain and nonbrain samples (*bottom*). Pink loci have effects of the same sign in both brain and nonbrain samples, even if the effect was only significant in the brain or nonbrain samples (general). Blue loci have effects with opposite signs in brain and nonbrain samples (pleiotropic). * indicates 2 linked, but distinguishable, loci on chromosome XII. c) The effects of loci detected in whole-genome scans using aggregate data are shown. Effects were calculated as the mean persistence of strains with the 3S allele at the focal locus minus the mean persistence of strains with the BY allele after correction for on-plate growth. The effect of each locus after correcting for on-plate growth is also shown.

We began determining the genetic basis of differences in persistence within and between organs. We performed linkage mapping on each of the samples with significant differences among segregants, detecting 494 loci in total (Fig. 3a and Supplementary Data 7 and 8). On average, 5.43 loci were identified per sample using permutations-based thresholds (min: 0, max: 12) and 90 of 91 samples had at least 1 detected locus that was also identified in another sample. Multiple loci were mapped in the spleen (181), liver (157), kidney (101), and brain (55), and many loci were detected in multiple samples (min = 2, max = 87, median = 4), as expected if segregants show reproducible phenotypes across samples due to a common set of loci. These detections could be consolidated to 35 distinct loci, based on overlapping confidence intervals (Supplementary Table 2). The number of loci identified in these samples showed a highly significant relationship with *H*^2^ (simple linear regression of number of loci on *H*^2^, *R*^2^ = 0.32, *P* = 4.02 × 10^−9^; Supplementary Fig. 5), suggesting heterogeneity in measurement noise across samples impacted statistical power. Also, the large number of tests performed in these scans could have resulted in false positives; there was no straightforward way to differentiate such false positives from true positives.

### Genetic mapping with aggregated data improves detection of loci

To improve statistical power and minimize the number of tests, we performed linkage mapping on aggregate brain and nonbrain measurements, as well as the difference between the 2 measurements; these measurements should be more precise than data from individual samples (Supplementary Data 9). The scans on brain, nonbrain, and difference measurements respectively identified 9, 9, and 10 loci (Fig. 3b). Some loci were mapped in multiple of these scans, resulting in the identification of 18 unique loci. Eight of these loci overlapped loci detected in controls, suggesting they affect growth and survival under multiple conditions (Fig. 3a–c and Supplementary Fig. 6). Loci detected in the brain, nonbrain, and brain versus nonbrain scans explained 89.7%, 62.3%, and 83.6% of *H*^2^ in their respective measurements.

The resolution of loci was poor, with confidence intervals from scans using aggregate data spanning 58 kb (min: 9 kb, max: 100 kb) and 32.5 genes (min: 6, max: 62) on average Supplementary Table 3. To better resolve these loci, we leveraged confidence intervals from detections of these loci in multiple individual samples. While average resolution was only slightly improved (mean interval = 24 kb, mean number of genes = 12, min number of genes = 2, max number of genes = 39; Supplementary Table 4), the 2 most finely resolved loci were each localized to 2 candidate protein-coding genes (Cherry *et al.* 2012). A locus on Chromosome XIV spanned a subunit of the BLOC-1 complex involved in endosomal maturation (*SNN1*) and a poorly understood, pleiotropic gene (*MKT1*) known to influence many quantitative traits in *S. cerevisiae* (Steinmetz *et al.* 2002; Demogines *et al.* 2008; Dimitrov *et al.* 2009; Lewis *et al.* 2014). A locus on Chromosome XV encompassed alcohol dehydrogenase (*ADH1*) and a gene regulated by phosphate levels (*PHM7*). Additionally, a locus on Chromosome XII fractionated into 2 distinct intervals, one including only the genes for DNA topoisomerase III (*TOP3*) and a thiamine transporter (*THI7*).

### Loci show a mix of general effects and antagonistic pleiotropy

**Fig. 4.**
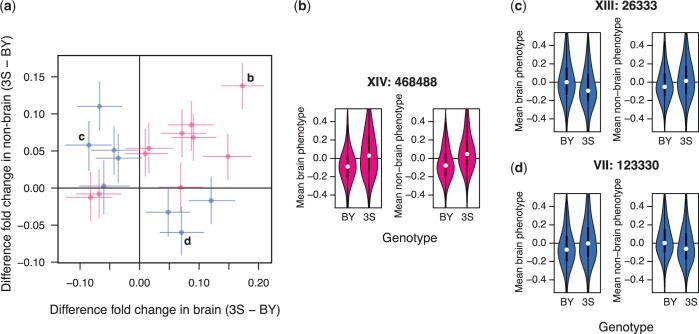
Identified loci show a mixture of general effects and antagonistic pleiotropy. a) The effect sizes of loci detected using aggregate phenotype data in the brain and nonbrain organs are shown on the x- and y-axes, respectively. Positive effect sizes mean that strains carrying the 3S allele were enriched in the samples while negative values mean that strains carrying the BY allele were enriched. Loci are colored by whether the same allele is beneficial in both brain and nonbrain samples (pink; general effects) or not (blue; antagonistic pleiotropy). Specific examples highlighted in panels (b)–(d). b) A locus with a general effect on persistence within the host. Brain (left) and nonbrain (right) phenotypes are plotted as a function of strain genotype at this locus. Positional information for the locus is denoted by bold text above the example. c) An antagonistically pleiotropic locus at which the BY allele is beneficial in the brain (left) and detrimental in other organs (right). d) An antagonistically pleiotropic locus at which the 3S allele is beneficial in the brain (left) and detrimental in other organs (right).

We next focused on understanding how the 18 loci identified in the scans with aggregate measurements influence the ability of segregants to persist in different parts of the mammalian body. We calculated the effects of each locus in the brain and nonbrain organs. Loci were then categorized as general or antagonistically pleiotropic if the same allele or different alleles were superior in the brain and nonbrain organs, respectively (Fig. 4). Specifically, the 2-dimensional space of persistence measurements in the brain and in nonbrain organs was split into 4 quadrants (Fig. 4a). Loci were classified based on whether their mean persistence measurement on each axis was above (>) or below (<) 0. Considering both brain and nonbrain organs, a locus could be ≫, ≪, ><, or <>. We defined loci with the ≫ and ≪ patterns, which indicated similar effects in different organs, as general effects (Fig. 4a lower left and upper right quadrants and Fig. 4b). We also defined loci with the >< and <> patterns, which showed opposing effects in different organs, as antagonistically pleiotropic (Fig. 4a lower right and upper left quadrants and Fig. 4, c and d).

Of the 18 loci, 10 were general and 8 were antagonistically pleiotropic. Eight of the beneficial alleles at the general loci were contributed by the 3S clinical isolate (Fig. 4a upper right quadrant), and 2 by the BY lab strain (Fig. 4a lower left quadrant). By contrast, both parental strains contributed alleles of the 8 antagonistically pleiotropic loci that were beneficial in either the brain or in nonbrain organs. Among the antagonistically pleiotropic loci, the BY strain contributed 5 alleles that were beneficial in the brain (Fig 4a, upper left quadrant) and 3 alleles that were beneficial in nonbrain organs (Fig 4a, lower right quadrant). Among these same antagonistically pleiotropic loci, the 3S strain contributed 3 alleles that were beneficial in the brain (Fig 4a, lower right quadrant) and 5 alleles that were beneficial in the nonbrain organs (Fig 4a, upper left quadrant).

### Identified loci collectively explain segregant persistence in different organs

**Fig. 5.**
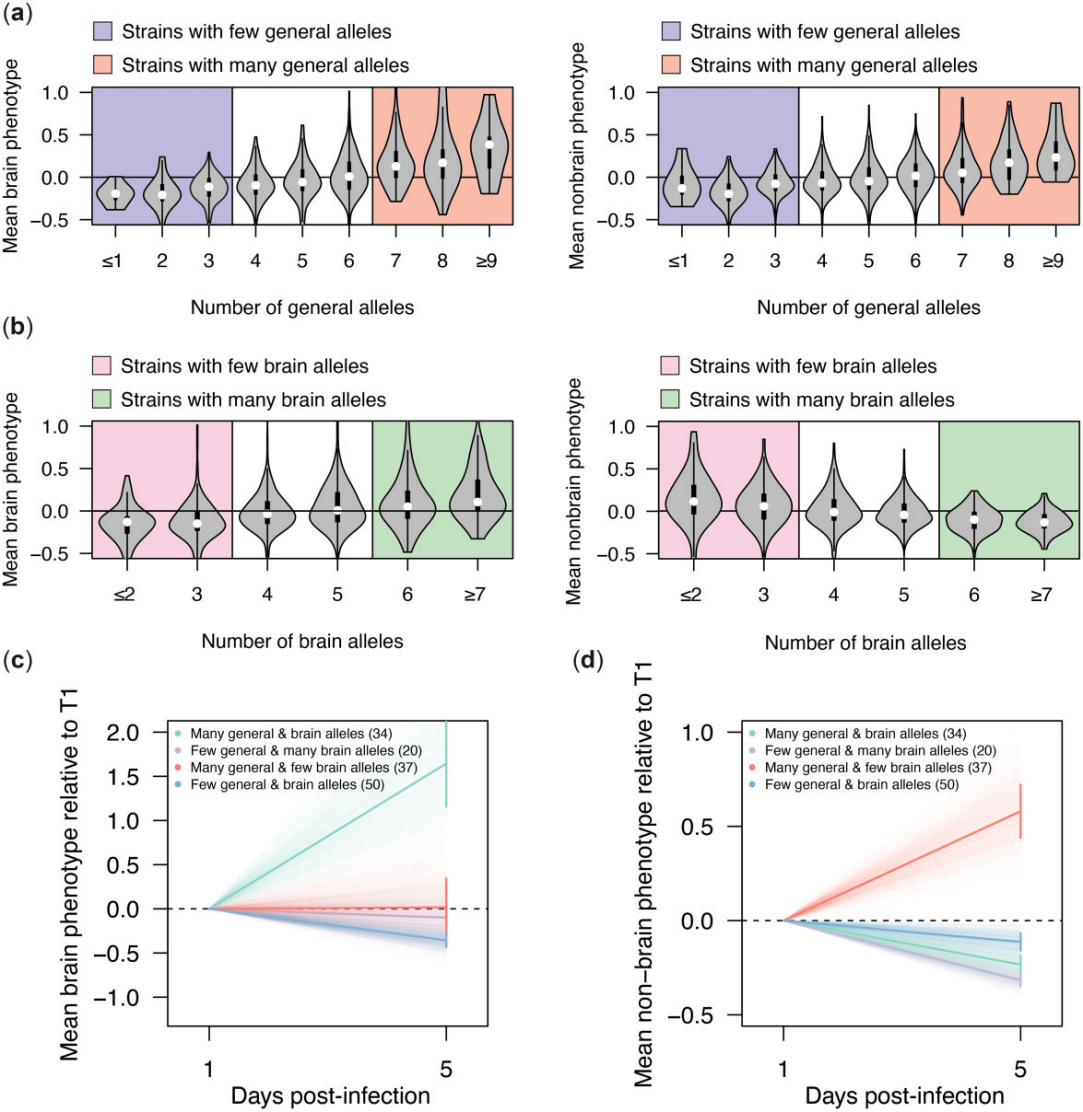
General and antagonistically pleiotropic loci collectively influence strain persistence in the host. a) Violin plots showing the mean strain phenotypes in the brain samples (left) and nonbrain samples (right) as a function of the number of generally beneficial alleles present in a segregant. Thresholds for strains considered to have a high or low number of general persistence alleles are represented by colored backgrounds. b) Violin plots showing the mean strain phenotypes in the brain (left) and nonbrain samples (right) as a function of the number of antagonistically pleiotropic brain alleles present in a strain. Thresholds for strains considered to have a higher or low number of alleles favoring persistence in the brain over other organs are represented by colored backgrounds. c) Plot showing mean change in enrichment in the brain samples over time relative to *T*_1_ measurements (bold lines) for strains that have a high or low number of generally beneficial alleles as well as a high or low number of alleles favoring persistence in the brain over other organs (according to thresholding in panels a and b). Error bars show the standard error about the mean enrichment of strains at 5 days postinfection. Faint lines show the enrichment over time of bootstrapped data (1,000 replicates). d) Plot showing mean enrichment in the nonbrain samples over time relative to day 1 measurements (bold lines) for strains that have a high or low number of generally beneficial alleles as well as a high or low number of alleles favoring persistence in the brain over other organs (according to thresholding in panels a and b). Error bars show the standard error about the mean enrichment of strains at 5 days postinfection. Faint lines show the enrichment over time of bootstrapped data (1,000 replicates).

We also determined how alleles at general and antagonistically pleiotropic loci combine to cause fungal persistence. There was a positive relationship between the number of beneficial alleles at general loci and persistence in both brain and nonbrain organs (simple linear regression of number of general alleles on persistence, brain *R*^2^ = 0.20 and *P* = 6.45 × 10^−41^, nonbrain *R*^2^ = 0.11 and *P* = 8.12 × 10^−23^; Fig. 5a). Similarly, we found that the number of brain or nonbrain alleles at antagonistically pleiotropic loci was positively and negatively related to segregants’ persistence in the brain (simple linear regression of number of brain alleles on brain persistence, *R*^2^ = 0.12, *P* = 1.32 × 10^−23^) and nonbrain organs (simple linear regression of brain alleles on nonbrain persistence, *R*^2^ = 0.08, *P* = 2.51 × 10^−16^), respectively (Fig. 5b).

Lastly, we examined how sets of general and antagonistically pleiotropic loci jointly influence persistence. In a given organ, the most persistent segregants were enriched for both the beneficial alleles at general loci and the appropriate alleles at antagonistically pleiotropic loci (brain 2 × 2 χ^2^ test: χ^2^ = 21.89, *P* = 2.9 ×10^−6^; nonbrain 2 × 2 χ^2^ test: χ^2^ = 15.42, *P* = 8.6 × 10^−5^; Fig. 5, c and d). In the brain, segregants were even able to persist if they were enriched for organ-specific alleles alone, although their persistence was lower than segregants enriched for both organ-specific and generally beneficial alleles (Fig. 5c).

## Discussion

We used barcode sequencing to phenotype a pool of genotyped haploid *MATɑ* segregants in mice. Analysis of segregants replicated in the pool showed that persistence in mice has a largely genetic basis, and comparison of samples revealed different segregants are superior in the brain and in the kidneys, liver, and spleen. Although technical noise limited our ability to map many loci in individual samples, aggregating brain and nonbrain samples made it possible to identify loci explaining most of the variability in persistence within and between organs. A total of 18 loci were detected, with the majority having effects across all organs. Some of these loci were generally beneficial, while others exhibited antagonistic pleiotropy, showing tradeoffs between brain and nonbrain organs. These antagonistically pleiotropic loci could represent either single polymorphisms that have different effects in distinct parts of the host body or closely linked polymorphisms with different effects.

Our findings may explain why diverse *S. cerevisiae* isolates act as opportunistic pathogens (Liti *et al.* 2009; Strope *et al.* 2015). The ability to persist in mammalian hosts is highly polygenic: we identified 18 loci in a cross of 2 isolates and examination of additional isolates would likely detect even more (Ehrenreich *et al.* 2012; Bloom *et al.* 2019). With so many loci involved, many *S. cerevisiae* isolates will possess beneficial alleles at some general loci, as we saw with both BY, an avirulent isolate (Clemons *et al.* 1994), and 3S, a clinical isolate (Liti *et al.* 2009). Furthermore, all isolates will carry alleles of antagonistically pleiotropic loci that are beneficial somewhere in the host body. Thus, the mixing of genetic material throughout the species by outcrossing may by chance produce strains that can persist in particular mammalian organs. Supporting such a possibility, clinical isolates are often strains possessing genetic signatures of recent outcrossing in nature, including high heterozygosity (Magwene *et al.* 2011; Strope *et al.* 2015).

Our results, in particular the identification of numerous antagonistically pleiotropic loci, also indicate that different organs in the mammalian body represent distinct environments for fungi. The brain and nonbrain organs have a myriad of functional and physiological differences: for example, the brain has its own semipermeable barrier (Daneman and Prat 2015; Profaci *et al.* 2020) and the kidneys, liver, and spleen filter blood (The Columbia Electronic Encyclopedia 2000). Persisting in these distinct organs may require different traits, which may be beneficial in some organs and detrimental in others. If these traits vary across strains, which seems likely based on our data, many yeast cells may only be able to infect certain organs in the mammalian body and may be constrained in their potential to spread to other organs postinfection.

Future work should examine whether the loci identified in our study are influenced by haploid mating type and ploidy, which both affect how diverse fungal pathogens interact with hosts (Morrow and Fraser 2013; Gerstein *et al.* 2017; Usher 2019). In the current work, we only examined *MATα* haploid segregants. We included a single haploid mating type in the pool to ensure strains did not mate within hosts, which would have disrupted the linkage between barcodes and genotypes, and to eliminate dominance among alleles at individual loci, which reduces the statistical power of linkage mapping. However, a drawback of our experimental design was that we could not assess whether identified loci show different effects in *MATa* haploid or *MATa/MATα* diploid segregants. Additionally, the present study does not provide insight into the phenotypes of diploids produced by mating haploids showing persistence in the brain and in nonbrain organs. In the future, such diploids should be generated and phenotyped for persistence, as it is possible they might be able to persist throughout the host body.

In addition to haploid mating type and ploidy, our study has additional limitations. The segregants we utilized were *ura3Δ*, an auxotrophy that has been reported to reduce yeast persistence in mice (Goldstein and McCusker 2001). However, it is possible the *ura3Δ* mutation may have aided our study by causing less persistent segregants to be more easily eliminated by their mouse hosts, thereby enhancing phenotypic differences among segregants. In such a scenario, the *ura3Δ* mutation might have in fact increased the statistical power of linkage mapping. Future work comparing *URA3* and *ura3Δ* versions of the cross should interrogate the costs and benefits of employing the *ura3Δ* marker in the context of genetic mapping studies in hosts. Furthermore, we did not evaluate the contribution of mitochondrial genotype, which can impact diverse traits in yeast (Dimitrov *et al.* 2009; Paliwal *et al.* 2014; Wolters *et al.* 2018; Vijayraghavan *et al.* 2019). Future work should also evaluate the interplay between mitochondrial genotype and the genetic factors identified in the present study.

Genetic mapping has the potential to help reveal molecular mechanisms shaping the abilities and constraints of persistence in different parts of the host body. Although our resolution was coarse in most cases, a few finely resolved loci implicated a potential diversity of cellular processes, including endosome maturation (*SNN1*), ethanol production (*ADH1*), genome stability (*TOP3*), phosphate metabolism (*PHM7*), and thiamine uptake (*THI7*). The other gene in these intervals (*MKT1*) has an unclear function. Notably, *ADH1* (Song *et al.* 2019), *MKT1* (Son *et al.* 2019), and *PHM7* (Jiang and Pan 2018) have been found to affect pathogenicity in other fungi, and both endosomal function (Bercusson *et al.* 2017) and thiamine transport (Huang *et al.* 2019) have been linked to virulence as well. Our system provides an opportunity not only to identify new mechanisms underlying persistence in hosts, but also to study how both known and unknown mechanisms act in combination, especially if higher-resolution mapping strategies are employed. Any such higher-resolution strategies will need to be compatible with the limited number of yeast that can be recovered from the brain.

Finally, fungal infections in the brain and central nervous system (meningitis) are a leading cause of morbidity and mortality among immunocompromised patients (Uppin *et al.* 2011; Kohler *et al.* 2014). We detected specific allele combinations that allowed segregants to persist in the brain, but our limited mapping resolution precluded insight into how these alleles act mechanistically. A possibility is they influenced passage through the blood–brain barrier, as we recovered fewer yeast from the brain than the kidneys, liver, or spleen. This hypothesis, which requires future testing, illustrates how our experimental system can be used to understand the mechanisms by which genetic polymorphisms modify interactions between yeast cells and the mammalian body.

## Supplementary Material

iyac138_Supplementary_Data

## Data Availability

All strains and data are available upon request. Processed data used in the article are included in the Supplementary Materials. Raw genotyping and barcode sequencing data are available through the NCBI Sequence Read Archive Bioproject PRJNA856040. Supplemental material is available at GENETICS online.
