# Supplementary material for: Complex genetics cause and constrain fungal persistence in different parts of the mammalian body
Source: Genetics. 2022 Sep 14;222(3):iyac138. doi: 10.1093/genetics/iyac138 (PMC9630980; doi:10.1093/genetics/iyac138)
Supplement: iyac138_Supplementary_Data [file iyac138_supplementary_data.zip › Supplemental_Material_GENETICS-2022-305517.pdf]

Supplemental information for

Complex genetics cause and constrain fungal persistence in different parts of the mammalian body

Martin N. Mullis, Caleb Ghione, Michael Lough-Stevens, Ilan Goldstein, Takeshi Matsui, Sasha F. Levy, Matthew D. Dean, Ian M. Ehrenreich

Supplementary figures

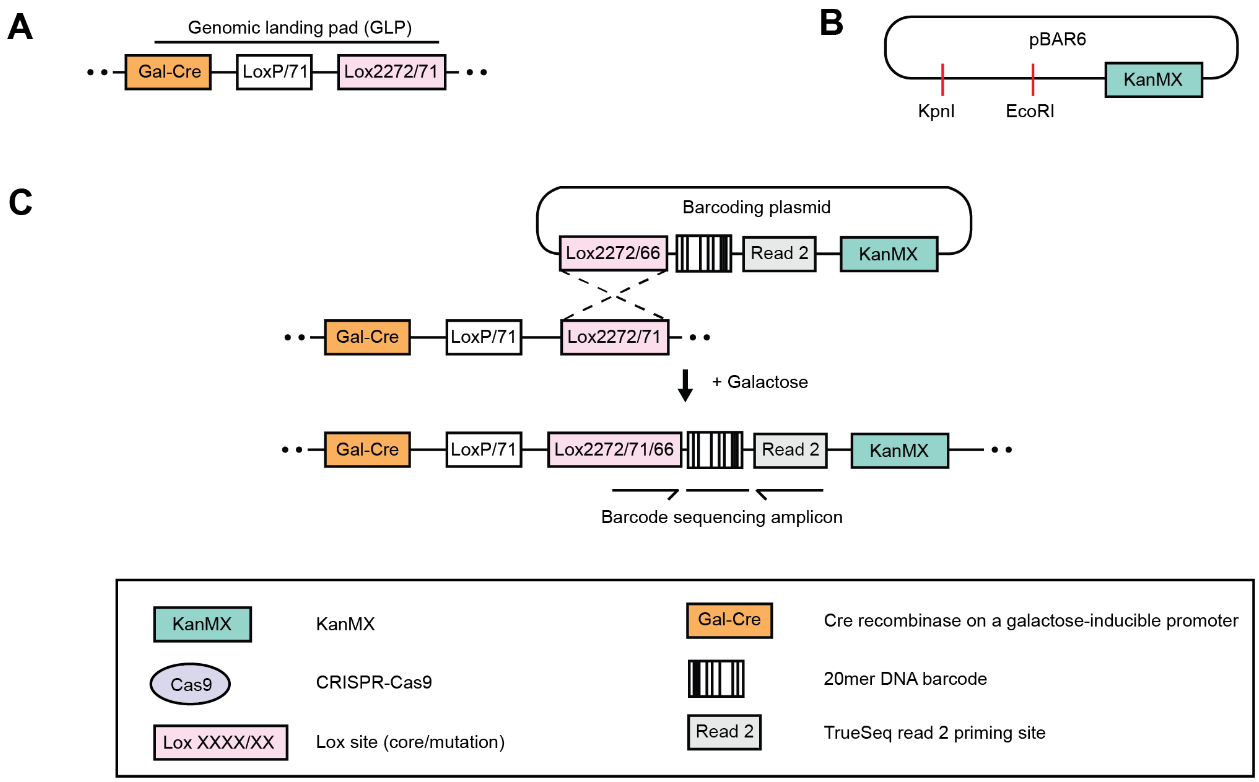

Figure S1. Barcoding of haploid segregants. a, The parental strains used to generate haploid segregants for this study were each transformed with a genomic landing pad consisting of a galactose-inducible Cre recombinase and partially crippled LoxP sites. This construct was inserted at the neutral *YBR209W* site. **b,** The plasmid pBAR6 was used to generate a barcode library for integration into haploid segregants. This plasmid contains a KanMX marker. **c,** Barcoding plasmids were constructed via Gibson assembly of linearized pBAR6 with a PCR product containing a partially crippled Lox2272/66 site, a random 20-mer barcode sequence, and a partial TruSeq read 2 adapter sequence. This plasmid library was then transformed individually into each segregant. Galactose was used to induce Cre-Lox recombination between barcoded plasmids and the genomic landing pad at Lox 2272, resulting in integration of the barcode into the genome of each segregant. Segregants were plated on YPD containing G418 to select for integration.

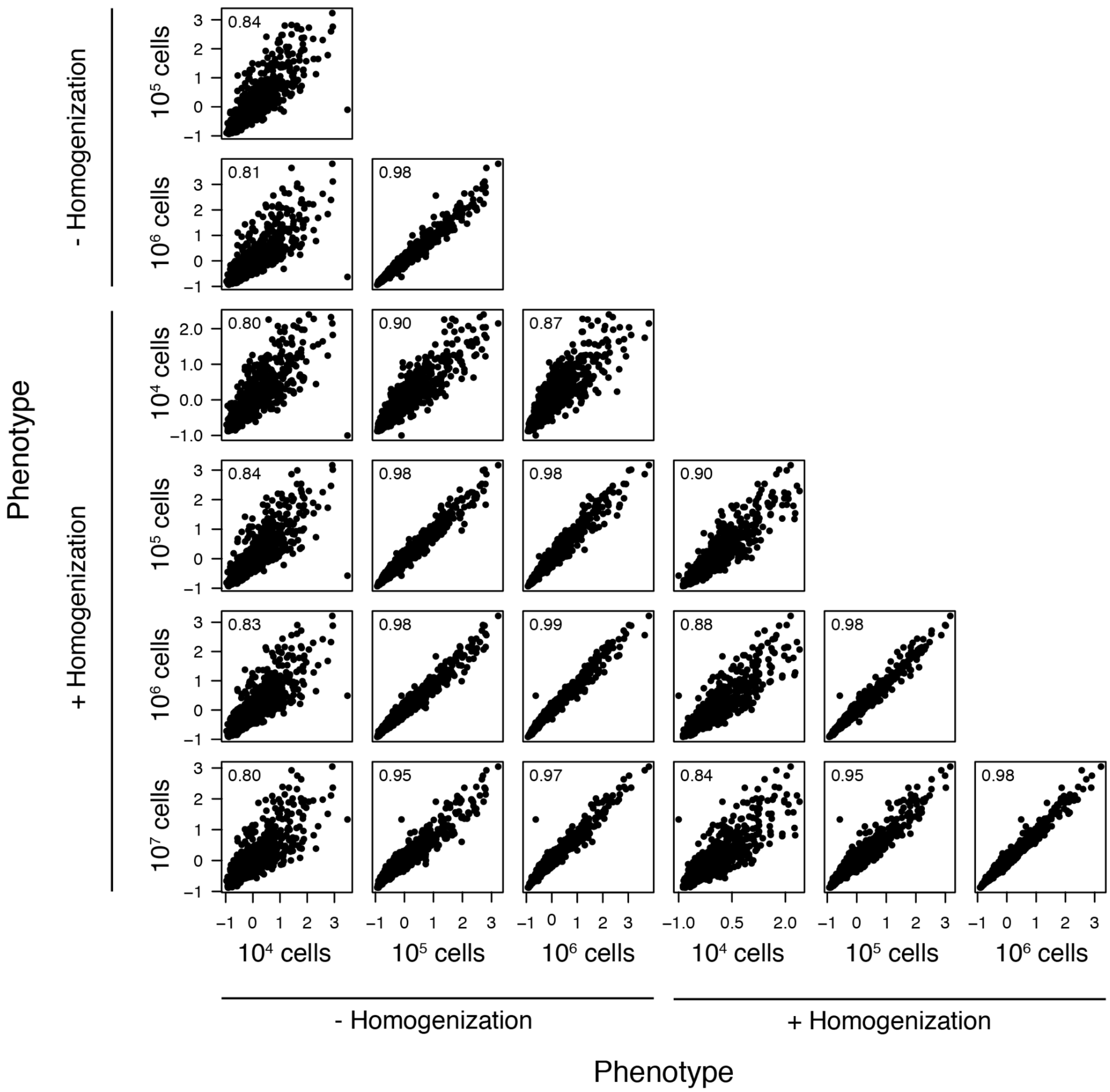

Figure S2. Reproducibility of controls across plating density and organ homogenization. a, Scatterplots showing all pairwise comparisons of samples that underwent the tissue homogenization process prior to plating (+ Homogenization) or not (- Homogenization). Each dot represents a different segregant. Phenotypes are mean phenotypes from 2 to 3 technical replicate experiments with the same treatment and plating density. The Pearson correlation coefficient for each pairwise comparison is in the upper-left corner of each plot.

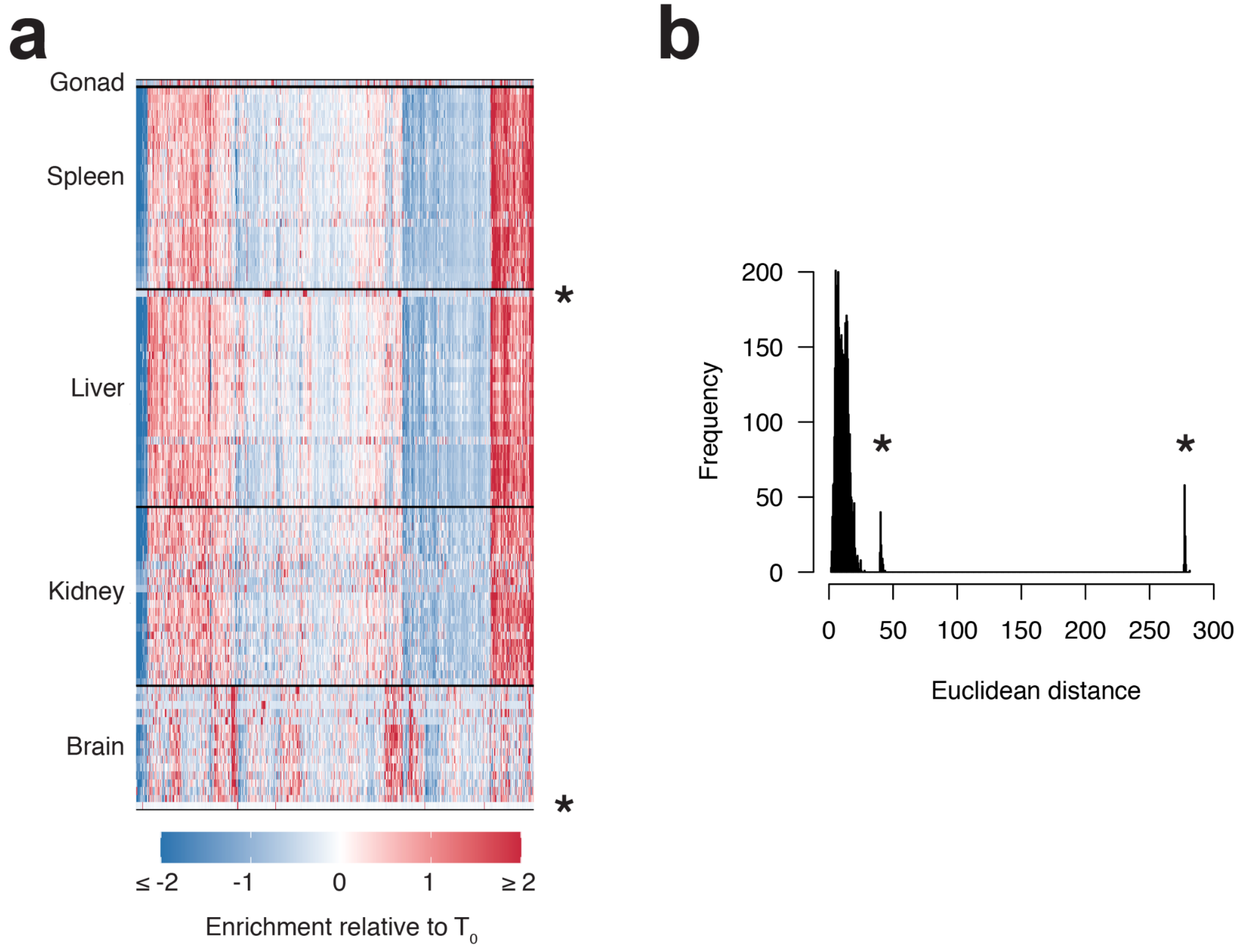

Figure S3. Outlier brain, gonad, and liver samples. a, Phenotypes of segregants (x-axis) are shown across organ samples (y-axis). Two outlier samples are marked by * symbols. These samples were poorly correlated with others in the dataset, contained many segregants with near-zero barcode counts, and had unusually high phenotypic variance among segregants. These outliers likely reflect poor recovery of yeast from these samples, poor quality sequencing library preparations, or a combination of the two. Thus, these outlier brain and spleen samples were excluded from downstream analyses. The single gonad sample was also excluded from downstream analyses due to a lack of within-organ replication and the fact that the gonad had similar properties to the two outlier samples. **b,** Histogram of euclidean distances between all pairs of the 94 organ samples prior to exclusion of the gonad and outlier samples. Distances including one or more of the three excluded samples are marked by * and clearly show much higher distances than all other sample-sample comparisons.

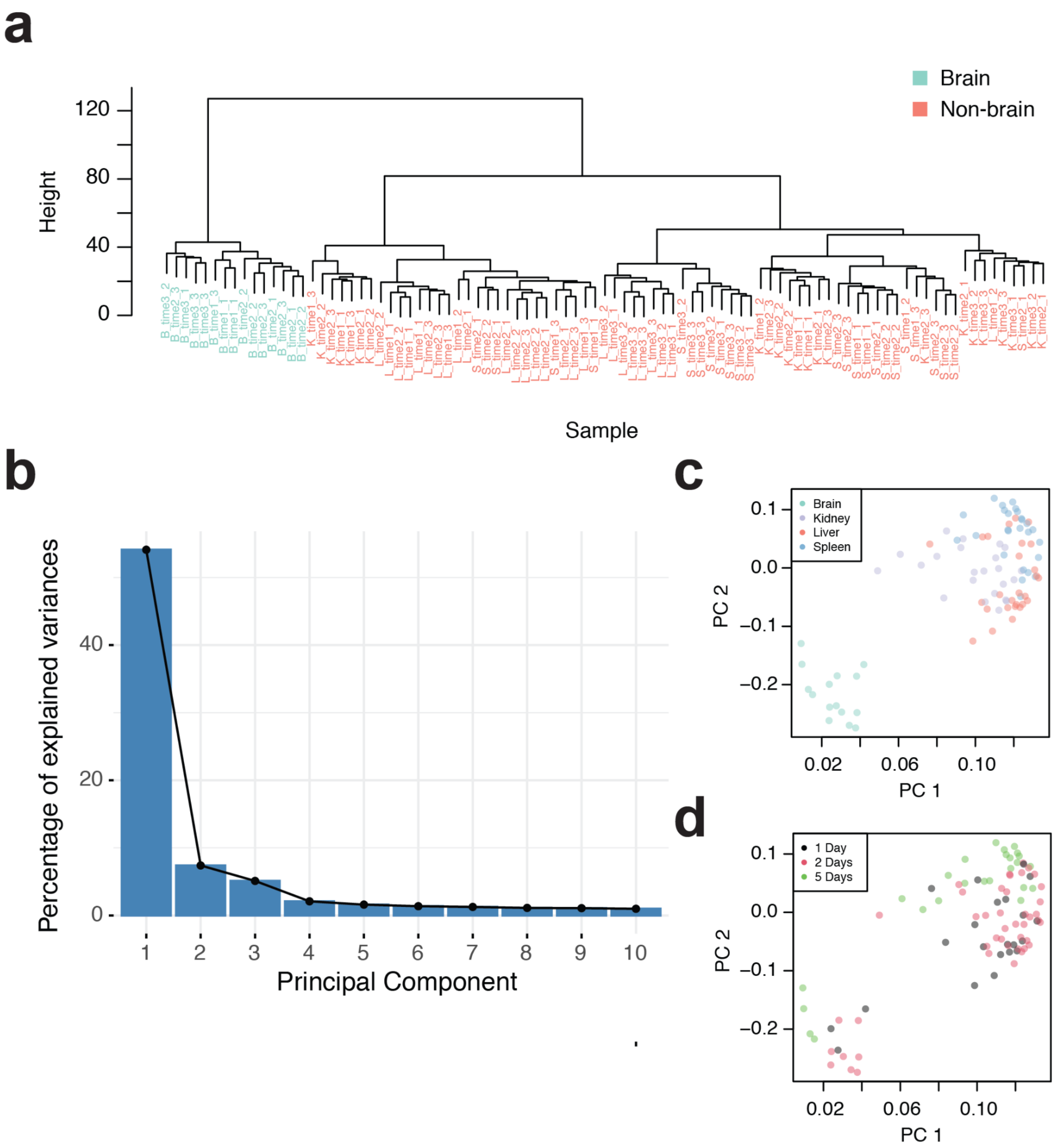

Figure S4. Hierarchical clustering and principal components analysis of organ samples. a, Dendrogram showing the relationships among hierarchically clustered organ samples. The two main groups are colored cyan (brain) and orange (non-brain). Sample labels include organ, time, and replicate information, separated by underscores. **b,** Scree plot showing the percentage of variance across organ samples explained (y-axis) by each principal component (x-axis). The first principal component explains most variability in the dataset. **c,** Sample loadings on the first two principal components. Samples are colored by organ type. The main division captured by the first principal component is brain vs. non-brain. **d,** Sample loadings on

the first two principal components. Samples are colored according to the time point at which they were collected. The second principal component captures a time effect that is much weaker than the brain vs. non-brain effect.

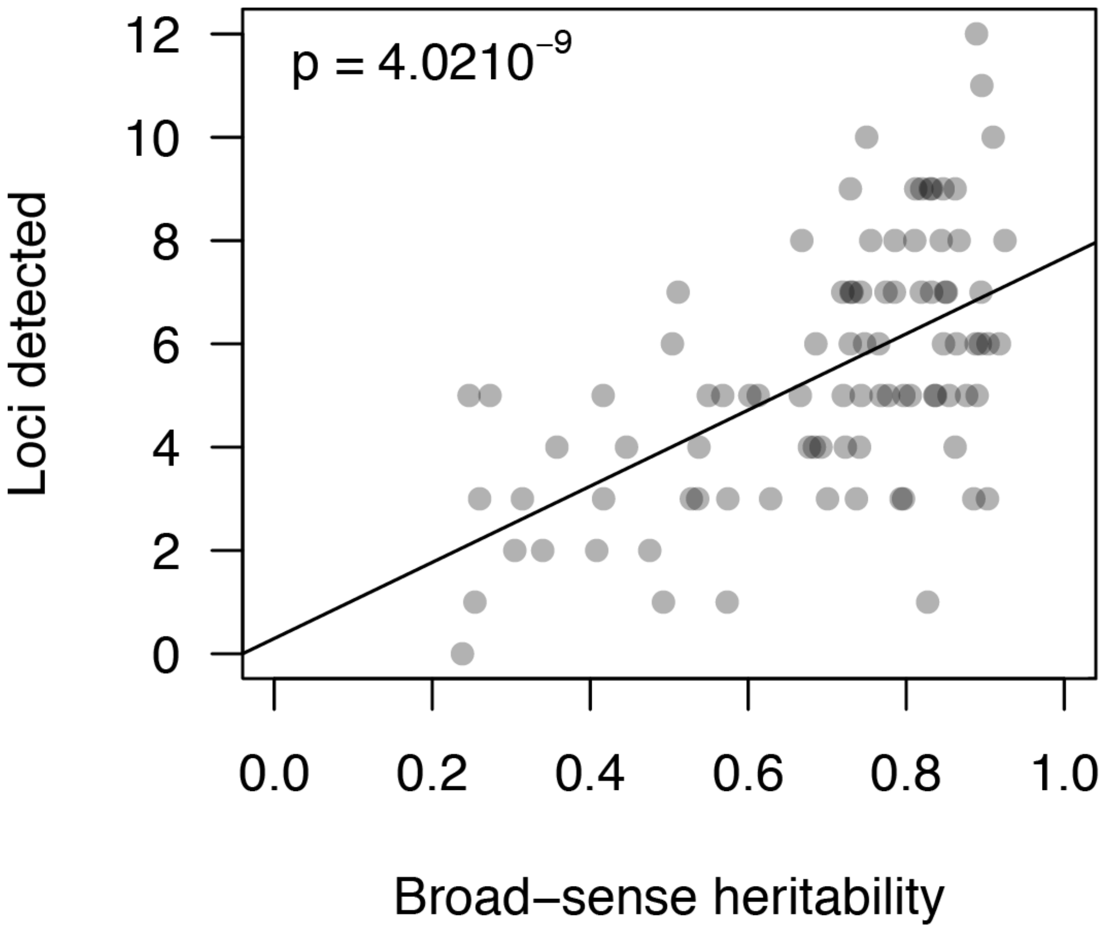

Figure S5. Samples show a positive relationship between broad-sense heritability and the number of detected loci. Scatterplot of the number of loci detected in samples as a function of their broad-sense heritabilities.

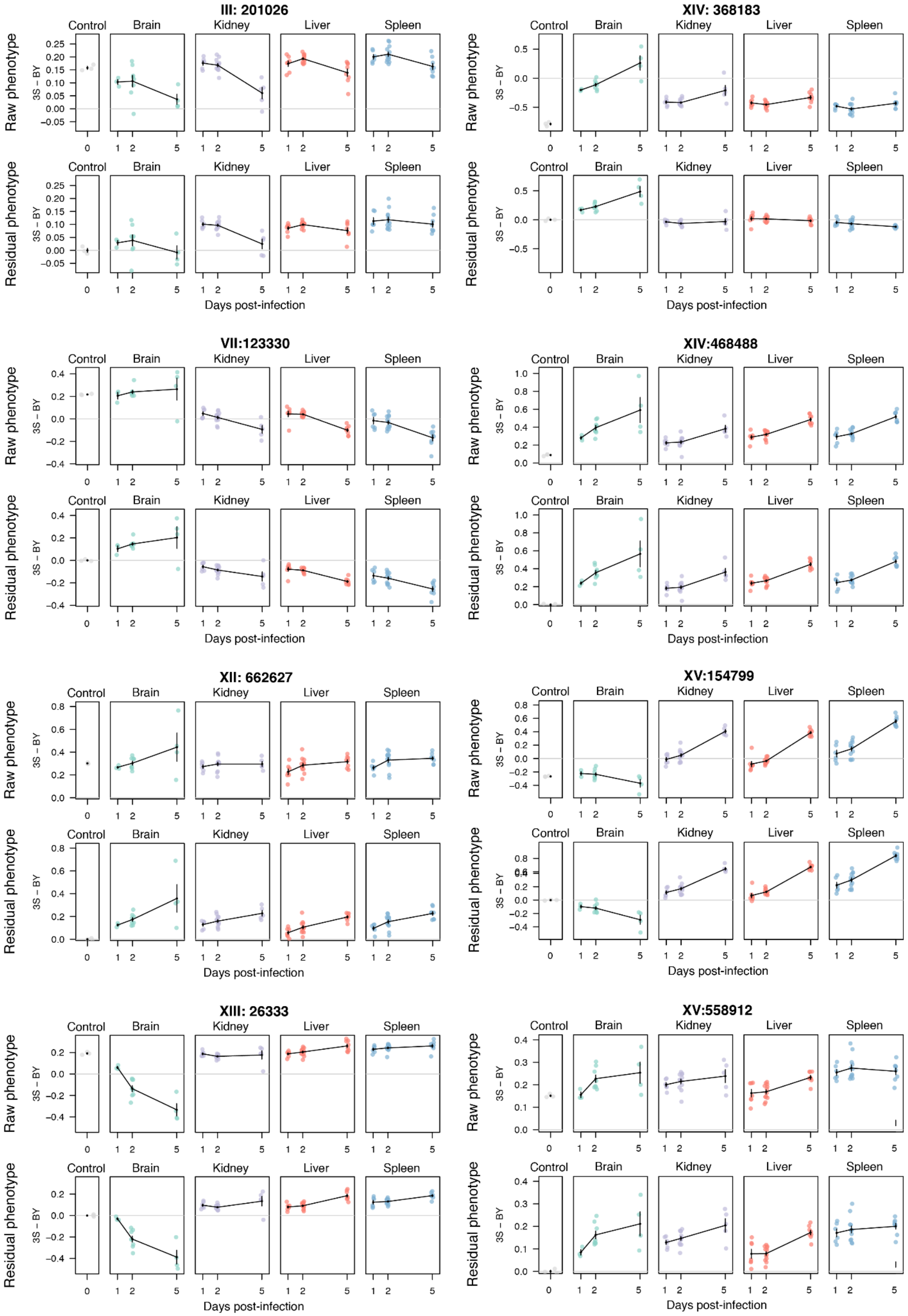

III: 201026
XIV: 368183
VII:123330
XIV:468488
XII: 662627
XV:154799
XIII: 26333
XV:558912
Control
Brain
Kidney
Liver
Spleen
Raw phenotype
Residual phenotype
3S – BY
Days post-infection

Figure S6. Loci detected in both control and organ samples show different effects in these two contexts. Data are shown for eight loci detected in both on-plate control samples and aggregate data. Effects were calculated as the mean persistence of strains with the 3S allele at the focal locus minus the mean persistence of strains with the BY allele. For each locus, the effect sizes of the locus across sample types before correction for on-plate growth ("Raw phenotype", top row) and after correction for on-plate growth ("Residual phenotype", bottom row) are shown. In the paper, all mapping results for organ samples were generated using the latter. In all cases, the loci showed different effects in the organ samples than the control samples.

PC	Eigenvalue	Percent variance explained	Cumulative percent variance explainedt
1	49.2313938	54.1004327	54.1004327
2	6.7249852	7.39009363	61.4905263
3	4.64572989	5.10519768	66.595724
4	1.90195534	2.09006081	68.6857848
5	1.45642361	1.60046551	70.2862503
6	1.24398835	1.36702017	71.6532705
7	1.1446708	1.25788	72.9111505
8	1.01284261	1.11301386	74.0241644
9	0.97674384	1.07334487	75.0975092
10	0.89437642	0.98283123	76.0803405

Table S1. Results from principal component analysis. Eigenvalues and variance explained by the first ten principal components (PCs) across the 91 samples with significant differences in persistence among strains. Each principal component is listed in column 1 ("PC"). The eigenvalue of each PC is listed in column 2 ("Eigenvalue"). The percentage of variance in the data explained by each PC is listed in column 3 ("Percent variance explained"). Column 4 tracks the cumulative variance explained by each PC and all preceding PCs ("Cumulative percent variance explained").

Chromosome	Position	-log10(p-value)	Samples with detection	CI start	CI end
14	467028	31.9055081	87	466103	468488
15	163253	26.596455	55	159655	165358
10	656567	14.1894071	17	655475	664824
14	382896	13.7777158	45	373134	388136
15	558912	12.9211021	26	552421	569690
12	677900	12.1338515	13	648649	680005
13	27865	12.0857298	19	23620	29994
15	506101	8.78892383	12	502258	512566
12	614136	8.31693972	46	611179	614708
12	604895	7.66591787	2	603024	609791
15	86817	7.58198738	6	79585	91150
2	514806	7.18993782	4	503196	536637
13	333449	7.17131203	15	327769	341722
15	328791	7.16382027	7	289957	341179
8	113931	7.10793557	16	96126	117504
7	141481	6.67391166	41	120263	144867
13	369062	6.53474435	3	352789	384682
13	50831	6.11920421	4	45801	65881
2	247993	6.00542966	3	215181	254349
3	195779	5.92591648	19	190765	209941
3	162694	5.91797534	3	137654	163810
8	422538	5.76723709	10	413653	465091
11	531524	5.01907183	2	527638	557824
12	82033	4.9644584	3	70019	91612
10	38691	4.94663669	2	24374	41794
4	694033	4.88346484	7	671424	703386
4	484028	4.73426903	2	453755	520815
4	1420155	4.6805821	2	1398006	1466633
13	923989	4.67297161	2	904006	923989
11	210267	4.66220151	3	193550	214537
12	854278	4.63793522	3	837919	872180
6	173376	4.60662207	2	167198	201882
8	218718	4.58254191	2	185403	242447
7	444466	4.55021075	2	396575	483704
16	530286	4.5376581	2	484429	581662

Table S2. Loci detected across individual organ samples. Loci detected in individual organ samples, consolidated by overlapping confidence intervals. Chromosome and positional information for each locus are listed in columns 1 (“Chromosome”) and 2 (“Position”). Column 3

lists the maximum -$\log_{10}$(p value) observed across samples for each locus (“-log10(p-value)”). Column 4 lists the number of samples each locus was detected in (“Samples with detection”); only reproducible loci detected in at least two samples are listed. The minimal confidence intervals observed across all consolidated detections are listed in columns 5 and 6 (“CI start” and “CI end”, respectively).

Chromosome	Position	Brain	NonBrain	B vs NB	CI start	CI end
14	468488	1	1	0	464233	473648
15	154799	1	1	1	151709	164284
10	662146	1	0	1	654612	665450
13	26333	1	1	1	13364	32621
14	368183	0	1	1	366896	390845
7	123330	0	1	1	95130	146193
12	604895	0	1	0	601489	622969
12	662627	1	0	0	629690	721983
4	505548	1	0	0	453843	540228
4	1425361	0	0	1	1420155	1507540
15	558912	1	1	0	516828	604757
3	201026	0	1	0	109046	210757
2	499454	1	0	0	434976	517160
13	329249	0	0	1	319136	346162
8	74372	0	1	0	56225	121745
4	148638	1	0	1	131075	235676
8	422538	0	0	1	380512	451071
5	481570	0	0	1	470796	560376

Table S3. Loci detected in aggregate scans. Loci detected in scans using aggregated phenotypes, consolidated by overlapping 95% (2LOD) confidence intervals. Chromosome and positional information for each locus are listed in columns 1 (“Chromosome”) and 2 (“Position”). Whether the loci was detected (“1”) or not (“0”) in an aggregate scan using brain samples, non-brain samples, or the difference between mean brain and mean non-brain phenotypes is listed in columns 3 through 5 (“Brain, “NonBrain”, and “B vs NB”, respectively). The minimal confidence intervals observed across all consolidated detections are listed in columns 6 and 7 (“CI start” and “CI end”, respectively).

Chromosome	Position	Min interval	Max interval	Number of genes	Gene IDs
14	467028	466103	468488	2	YNL086W,YNL085W
15	163253	159655	165358	2	YOL086C,YOL084W
10	656567	655475	664824	4	YJR125C,YJR126C,YJR127C,YJR129C
13	27865	23620	29994	4	YML124C,YML123C,YML121W,YML120C
14	382896	373134	388136	10	YNL134C,YNL133C,YNL132W,YNL131W,YNL130C,YNL130C-A,YNL129W,YNL128W,YNL127W,YNL126W
7	141481	120263	144867	11	YGL201C,YGL200C,YGL198W,YGL197W,YGL196W,YGL195W,YGL194C-A,YGL194C,YGL193C,YGL192W,YGL191W
12	614136	611179	614708	2	YLR234W,YLR237W
12	604895	603024	609791	4	YLR229C,YLR231C,YLR233C,YLR234W
12	677900	648649	680005	14	YLR256W,YLR257W,YLR258W,YLR259C,YLR260W,YLR261C,YLR262C,YLR262C-A,YLR263W,YLR264W,YLR264C-A,YLR265C,YLR266C,YLR267W
4	484028	453755	520815	30	YDR003W,YDR003W-A,YDR004W,YDR005C,YDR006C,YDR007W,YDR009W,YDR011W,YDR012W,YDR013W,YDR014W,YDR014W-A,YDR016C,YDR017C,YDR018C,YDR019C,YDR020C,YDR021W,YDR022C,YDR023W,YDR025W,YDR026C,YDR027C,YDR028C,YDR030C,YDR031W,YDR032C,YDR033W,YDR034C,YDR034C-A
4	1420155	1398006	1466633	39	YDR468C,YDR469W,YDR470C,YDR471W,YDR472W,YDR473C,YDR475C,YDR476C,YDR477W,YDR478W,YDR479C,YDR480W,YDR481C,YDR482C,YDR483W,YDR484W,YDR485C,YDR486C,YDR487C,YDR488C,YDR489W,YDR490C,YDR492W,YDR493W,YDR494W,YDR495C,YDR496C,YDR497C,YDR498C,YDR499W,YDR500C,YDR501W,YDR502C,YDR503C,YDR504C,YDR505C,YDR506C,YDR507C,YDR508C
15	558912	552421	569690	8	YOR122C,YOR123C,YOR124C,YOR125C,YOR126C,YOR127W,YOR128C,YOR129C
3	195779	190765	209941	13	YCR034W,YCR035C,YCR036W,YCR037C,YC

					R038C,YCR039C,YCR040W,YCR041W,YCR042C,YCR043C,YCR044C,YCR045C,YCR046C
3	162694	137654	163810	15	YCR012W,YCR014C,YCR015C,YCR016W,YCR017C,YCR018C,YCR019W,YCR020C,YCR020C-A,YCR020W-B,YCR021C,YCR023C,YCR024C,YCR024C-B,YCR024C-A
2	514806	503196	536637	13	YBR133C,YBR135W,YBR136W,YBR137W,YBR138C,YBR139W,YBR140C,YBR141C,YBR142W,YBR143C,YBR145W,YBR146W,YBR147W
13	333449	327769	341722	9	YMR028W,YMR029C,YMR030W,YMR031C,YMR032W,YMR030W-A,YMR033W,YMR034C,YMR035W
8	113931	96126	117504	13	YHL007C,YHL006C,YHL004W,YHL003C,YHL002W,YHL001W,YHR001W,YHR001W-A,YHR002W,YHR003C,YHR004C,YHR005C,YHR005C-A
8	422538	413653	465091	23	YHR158C,YHR159W,YHR160C,YHR161C,YHR162W,YHR163W,YHR164C,YHR165C,YHR166C,YHR167W,YHR168W,YHR169W,YHR170W,YHR171W,YHR172W,YHR173C,YHR174W,YHR175W,YHR175W-A,YHR176W,YHR177W,YHR178W,YHR179W

Table S4. Candidate genes within loci detected in aggregate scans. Chromosome and positional information for each aggregate locus are listed in columns 1 (“Chromosome”) and 2 (“Position”). The minimal confidence intervals observed across all loci detected in individual samples that overlapped a particular aggregate locus are listed in columns 3 and 4 (“Min position” and “Max position”, respectively). The number of genes within each confidence interval is listed in column 5 (“Number of genes”). The gene IDs of all genes within a confidence interval are listed in column 6 (“Gene IDs”).

Data S1. Barcode data for the strains used in this study. Each row corresponds to a segregant present in the pool injected into mice at the beginning of the experiment. Column 1 contains the segregant name (‘Segregant’), while columns 2 through 4 contain information about the barcode(s) identifying that segregant in the pool (‘BC1’, ’BC2’, and ‘BC3’). ‘NA’ values indicate a barcode replicate did not exist.

Data S2. Colony counts from organ samples. Each row in the file contains information about one of the mice used in the study. This includes the identification number of the animal (‘ID’), the number of days post-inoculation at which the organ samples were collected (‘Time’), the sex of the animal (‘Sex’), and whether or not the animal was treated with dexamethasone (‘Treatment’). The numbers of colony forming units (CFU) observed from each organ sample collected from an animal are recorded in columns 5 - 9 of the file (‘Brain’, ‘Gonad’, ’Kidneys’, ‘Liver’, and ‘Spleen’).

Data S3. Barcode frequencies within each organ sample. Each row in the file contains barcode frequencies of all segregants used in the study within an individual organ sample. Frequencies were calculated by taking the raw counts of each barcode within a sample and dividing by the number of total barcodes observed in the sample. The first seven columns contain information about the organ sample and replicate to which the barcode frequencies correspond. Columns 1 through 4 contain information about the animal, including the identification number (‘id’), sex of the animal (‘sex’), the time point post-inoculation at which the organ sample was collected (‘time’), and whether the animal was treated with dexamethasone or saline (‘dex’). ‘time1’, ‘time2’, and ‘time3’ values in the ‘time’ column correspond to days 1, 2, and 5 post-inoculation, respectively., Column 5 (‘rep’) contains replicate information for each mouse, since three mice of each sex and treatment were used per time point. Column 6 (‘tissue’) contains information about the organ type for each sample, where each value corresponds to the first letter of the organ in which the barcode frequencies were measured. Column 7 (‘strainrep’) records the internal strain replicate being recorded in a particular row of the file, so that each replicate can be examined separately; as a result, there are three rows corresponding to each organ sample in the dataset. Columns 8 - 829 contain barcode frequency information for each segregant in the pool; columns are named by segregant.

Data S4. Mean persistence measurements in on-plate controls. Each row contains the mean persistence values of the first barcode replicate of all segregants across replicated control samples at a particular plating dilution and homogenization treatment. Column 1 contains information about which control the mean persistence values correspond to. ‘T4’, ‘T5’, ‘T6’, and ‘T7’ correspond to controls plated at densities of $1x10^4$, $1x10^5$, $1x10^6$, or $1x10^7$ cells per plate, respectively, after being passed through the tissue homogenization process. ‘No4’, ‘No5’, and ‘No6’ correspond to controls plated at $1x10^4$, $1x10^5$, or $1x10^6$ cells per plate directly, without being passed through the tissue homogenization process. The mean phenotype values from ‘T5’ were used to fit a linear model correcting for on-plate growth. Columns 8 - 829 contain mean persistence measurements for each segregant in a given control type; columns are named by segregant.

Data S5. Residual persistence measurements after correcting for on-plate growth. Each row in the file contains residual persistence measurements of all segregants used in the study within an individual sample after correcting for on-plate growth. As in the barcode frequency file, the first seven columns contain information about the organ sample and replicate to which the barcode frequencies correspond. Columns 1 through 4 contain information about the animal, including the identification number (‘id’), sex of the animal (‘sex’), the time point post-inoculation at which the organ sample was collected (‘time’), and whether the animal was treated with dexamethasone or saline (‘dex’). ‘time1’, ‘time2’, and ‘time3’ values in the ‘time’ column correspond to days 1, 2, and 5 post-inoculation, respectively. Column 5 (‘rep’) contains replicate information for each mouse, since three mice of each sex and treatment were used per time point. Column 6 (‘tissue’) contains information about the organ type for each sample, where each value corresponds to the first letter of the organ in which the barcode frequencies were measured. Column 7 (‘strainrep’) records the internal strain replicate being recorded in a particular row of the file, so that each replicate can be examined separately; as a result, there are three rows corresponding to each organ sample in the dataset. Values from strain replicate ‘1’ were used for linkage mapping in individual samples and aggregate brain/non-brain measurements. All three strain replicates were used to calculate heritability within samples. Columns 8 through 829 contain persistence measurements for each segregant in the pool after correcting for on-plate growth; columns are named by segregant.

Data S6. Broad-sense heritability and significance of genotype on persistence in individual organ samples. Columns 1 through 4 contain information about the each sample or control in the dataset, including the identification number (‘id’), sex of the animal (‘sex’), the time point post-inoculation at which the organ sample was collected (‘time’), and whether the animal was treated with dexamethasone or saline (‘dex’). ‘time1’, ‘time2’, and ‘time3’ values in the ‘time’ column correspond to days 1, 2, and 5 post-inoculation, respectively. ‘T4’, ‘T5’, ‘T6’, and ‘T7’ in the ‘id’ column correspond to controls plated at densities of $1x10^4$, $1x10^5$, $1x10^6$, or $1x10^7$ cells per plate, respectively, after being passed through the tissue homogenization process. ‘No4’, ‘No5’, and ‘No6’ in the ‘id’ column correspond to controls plated at $1x10^4$, $1x10^5$, or $1x10^6$ cells per plate directly, without being passed through the tissue homogenization process. All other ‘id’ values correspond to animals used in the study. Column 5 (‘rep’) contains replicate information for each mouse, since three mice of each sex and treatment were used per time point. Column 6 (‘tissue’) contains information about the organ type for each sample, where each value corresponds to the first letter of the organ in which the barcode frequencies were measured. Column 7 (‘H2’) contains broad-sense heritability estimates for each sample or control. Column 8 (‘H2e’) contains the standard error about each heritability estimate. Column 9 (‘p’) contains the p-value associated with the linear fixed-effects model *persistence ~ genotype + error* used to determine in which samples genotype significantly influenced persistence. Column 10 (‘CFU’) lists the number of colony forming units recovered from each sample.

Data S7. Genotype data for each segregant. Each row in the dataframe corresponds to a SNP used as a marker for genetic analysis. Columns 1 through 3 contain positional information for each marker. Column 1 (‘c’) lists the chromosome on which the marker is found. Column 2 (‘p’) lists the positional coordinate of the marker on its respective chromosome. Column 3 (‘gp’)

lists the cumulative position of the marker across all chromosomes (from 1 - 16) in the ~12 Mb genome. Columns 4 through 825 contain genotype data for each segregant across all markers used in this study. Genotype data is binarized, with a ‘0’ and ‘1’ corresponding to the ‘BY’ and ‘3S’ alleles, respectively.

Data S8. Loci detected in individual organ samples. Columns 1 (‘c’) and 2 (‘p’) contain chromosome and positional information about the peak marker of a locus detected in a particular sample. Column 3 (‘pval’) lists the -$\log_{10}$(p-value) of the peak marker for the locus. Column 4 (‘PVE’) lists the percent variance explained by this locus in the sample in which it was detected. Columns 5 (‘pmin’) and 6 (‘pmax’) contain the minimum and maximum positions of the 90% confidence interval about the detection. Columsn 7 - 11 contain information about the sample in which a locus was detected, including the mouse (‘id’), sex of the animal (‘sex’), the time point post-inoculation at which the organ sample was collected (‘time’), whether the sample was derived from an animal treated with dexamethasone or saline (‘dex’), and the organ type of (‘tissue’). ‘time1’, ‘time2’, and ‘time3’ values in the ‘time’ column correspond to days 1, 2, and 5 post-inoculation, respectively. Values in the ‘tissue’ column refer to the organ type of a sample by its first letter.

Data S9. Aggregate persistence measurements in brain and non-brain samples. Each row of the file corresponds to a segregant in the dataset. Columns 1 through 4 (‘Brain’, ’Kidney’, ‘Liver’, and ‘Spleen’) provide mean persistence measurements for segregants across samples of each organ type after correcting for time and on-plate growth. Column 5 (‘Non_brain’) contains mean persistence measurements for segregants across all non-brain samples. Column 6 (‘Diff_organ’) contains differences in mean brain and mean non-brain persistence for each segregant.
